# Postsynaptic Complexin Mediates Constitutive Exocytosis of Nicotinic Acetylcholine Receptor

**DOI:** 10.1002/advs.202520721

**Published:** 2026-03-26

**Authors:** Ya Wang, Yixiang Deng, Nan Xia, Shuzo Sugita, Shangbang Gao

**Affiliations:** ^1^ Key Laboratory of Molecular Biophysics of the Ministry of Education College of Life Science and Technology Huazhong University of Science and Technology Wuhan China; ^2^ Division of Experimental & Translational Neuroscience Krembil Brain Institute University Health Network Toronto Ontario Canada; ^3^ Department of Physiology Temerty Faculty of Medicine University of Toronto Toronto Ontario Canada

**Keywords:** ACR‐16, complexin, constitutive exocytosis, postsynaptic, UNC‐31/CAPS

## Abstract

Complexin is a small, soluble protein that binds SNARE complexes to both promote Ca^2^^+^‐triggered vesicle fusion and suppress spontaneous release at presynaptic terminals. In cultured hippocampal neurons, postsynaptic complexin has been implicated in activity‐dependent receptor exocytosis. Here, we identify a constitutive postsynaptic role of complexin at the *C. elegans* excitatory neuromuscular junction. Loss of CPX‐1—the major complexin homolog—markedly increases the amplitude of spontaneous miniature postsynaptic current (mPSC) in an extracellular Ca^2^^+^‐independent manner. This phenotype is rescued by postsynaptic, but not presynaptic, expression of CPX‐1. Moreover, disruption of nicotinic acetylcholine receptor subunit *acr‐16* abolishes the enhanced mPSC amplitude, and the endogenous expression levels of postsynaptic ACR‐16 are elevated in *cpx‐1* mutants. This process requires the dense‐core vesicle regulator UNC‐31/CAPS, as *unc‐31* null mutations eliminate both the increased mPSC amplitude and elevated ACR‐16 expression in *cpx‐1* mutants. In addition, CPX‐1 is also required for activity‐dependent exocytosis of postsynaptic ACR‐16. Together, these findings reveal that complexin regulates both constitutive and activity‐dependent exocytosis of postsynaptic receptors.

## Graphical abstract

Beyond its established presynaptic role, complexin is identified as a postsynaptic regulator of neurotransmitter receptor trafficking. By promoting constitutive and activity‐dependent exocytosis of nicotinic acetylcholine receptors, complexin controls spontaneous synaptic signaling at the C. elegans neuromuscular junction, revealing an unanticipated mechanism linking vesicle fusion proteins to postsynaptic receptor homeostasis.

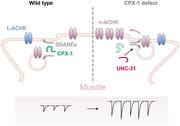

## 1 Introduction

Synaptic transmission relies on the tight coordination between presynaptic neurotransmitter release and the availability of postsynaptic receptors. This bidirectional interplay not only ensures reliable signal propagation, but also underpins circuit stability and activity‐dependent plasticity [1, 2, 3]. While presynaptic release mechanisms have been extensively dissected, the regulation of postsynaptic neurotransmitter receptors—their membrane‐localized levels, spatial distribution, constitutive and activity‐dependent trafficking—is equally fundamental for maintaining synaptic homeostasis and tuning synaptic strength [3, 4, 5, 6, 7, 8]. At peripheral synapses such as the neuromuscular junction, postsynaptic receptor exocytosis provides a key mechanism for sustaining receptor availability and balancing excitation and inhibition [9, 10, 11, 12, 13]. However, in contrast to the well‐characterized presynaptic secretion regulators, the molecular machinery that drives postsynaptic receptor exocytosis and trafficking remains far less defined.

Complexin (CPX) is a core component of the vesicle fusion machinery at presynaptic terminals. This small soluble protein promotes the assembly and stabilizes the neuronal Soluble N–ethylmaleimide sensitive factor attachment protein REceptor (SNARE) complex [14, 15, 16, 17, 18]. By regulating vesicular release across different vesicle pools, CPX ensures the temporal precision of synaptic transmission [19, 20]. A key mechanism is its ability to stabilize the partially assembled SNARE complex in a fusion‐ready state [18, 21, 22]. Through its dual actions, CPX enhances synchronous Ca^2^^+^‐evoked release while suppressing spontaneous release, thereby balancing fidelity and responsiveness at synapses [23, 24, 25, 26]. These functions of CPX are mediated by interactions with SNARE proteins and presynaptic calcium (Ca^2^^+^) sensors such as synaptotagmin‐1 [14, 21, 22, 27]. Across species, CPX function has diverged: in invertebrates such as *Caenorhabditis elegans* and *Drosophila*, CPX primarily suppresses spontaneous release [23, 25, 28], whereas in mammals, it predominantly facilitates spontaneous vesicle release [29, 30]. However, a commonality across both invertebrates and vertebrates, including specialized systems like bat auditory synapses, is that CPX facilitates evoked release [29, 30].

Beyond its established presynaptic function, complexin also exerts critical roles in postsynaptic exocytosis. In cultured hippocampal neurons, postsynaptic complexin selectively mediates activity‐dependent, but not constitutive, AMPA receptor insertion [31, 32]. Knockdown of complexin‐1/2 disrupts glycine‐induced AMPA receptor surface delivery—a hallmark of long‐term potentiation (LTP) [31, 33]. This pathway requires the Q‐SNARE proteins syntaxin‐3 and SNAP‐47, but notably bypasses its canonical presynaptic partner synaptotagmin‐1, pointing to a distinct postsynaptic fusion machinery [9, 31, 32]. Complexin is also required for the activity‐dependent dendritic release of brain‐derived neurotrophic factor (BDNF): loss of complexin‐1/2 abolishes theta‐burst‐induced BDNF secretion, a process instead mediated by synaptotagmin‐6 [34]. These pioneering findings identify a specialized postsynaptic function of complexin in regulating activity‐dependent receptor and neurotrophins exocytosis, underscoring its compartment‐specific roles across the synapse. However, most of this evidence is derived from in vitro systems, leaving unresolved the question of whether such mechanisms are conserved in vivo. If they are, does complexin require new or distinct molecular partners to regulate receptor secretion in vivo?

Here, we investigated the endogenous function of complexin at the *C. elegans* neuromuscular junction (NMJ), a genetically tractable model for dissecting synaptic transmission [35, 37]. Loss of CPX‐1, the *C. elegans* ortholog of mammalian complexin‐1, not only elevated spontaneous release frequency, but also markedly increased the amplitude of miniature postsynaptic currents (mPSCs) in a Ca^2^^+^‐independent manner. Compartment‐specific rescue experiments demonstrated that postsynaptic expression of CPX‐1 was sufficient to restore mPSC amplitude to wild‐type levels. This effect required the muscle‐specific nicotinic acetylcholine receptor subunit ACR‐16, indicating that CPX‐1 regulates constitutive exocytosis of nicotinic acetylcholine receptors (n‐AChRs) at postsynaptic sites. Indeed, muscular ACR‐16 was significantly elevated in *cpx‐1* mutant. Strikingly, CPX‐1 mediated ACR‐16 exocytosis required UNC‐31/CAPS, an evolutionarily conserved Ca^2^^+^‐binding protein we previously characterized as a presynaptic complexin partner [38]. CPX‐1 also contributed to activity‐dependent exocytosis of ACR‐16, suggesting that it coordinates both constitutive and regulated receptor exocytosis. Together, these findings reveal a previously unrecognized in vivo role for postsynaptic complexin in controlling n‐AChRs exocytosis, providing new insight into the conserved molecular logic of complexin‐dependent exocytosis across synaptic compartments.

## 2 Results

### 2.1 *cpx‐1* Mutants Display Increased Amplitude of Spontaneous Release

**FIGURE 1:**
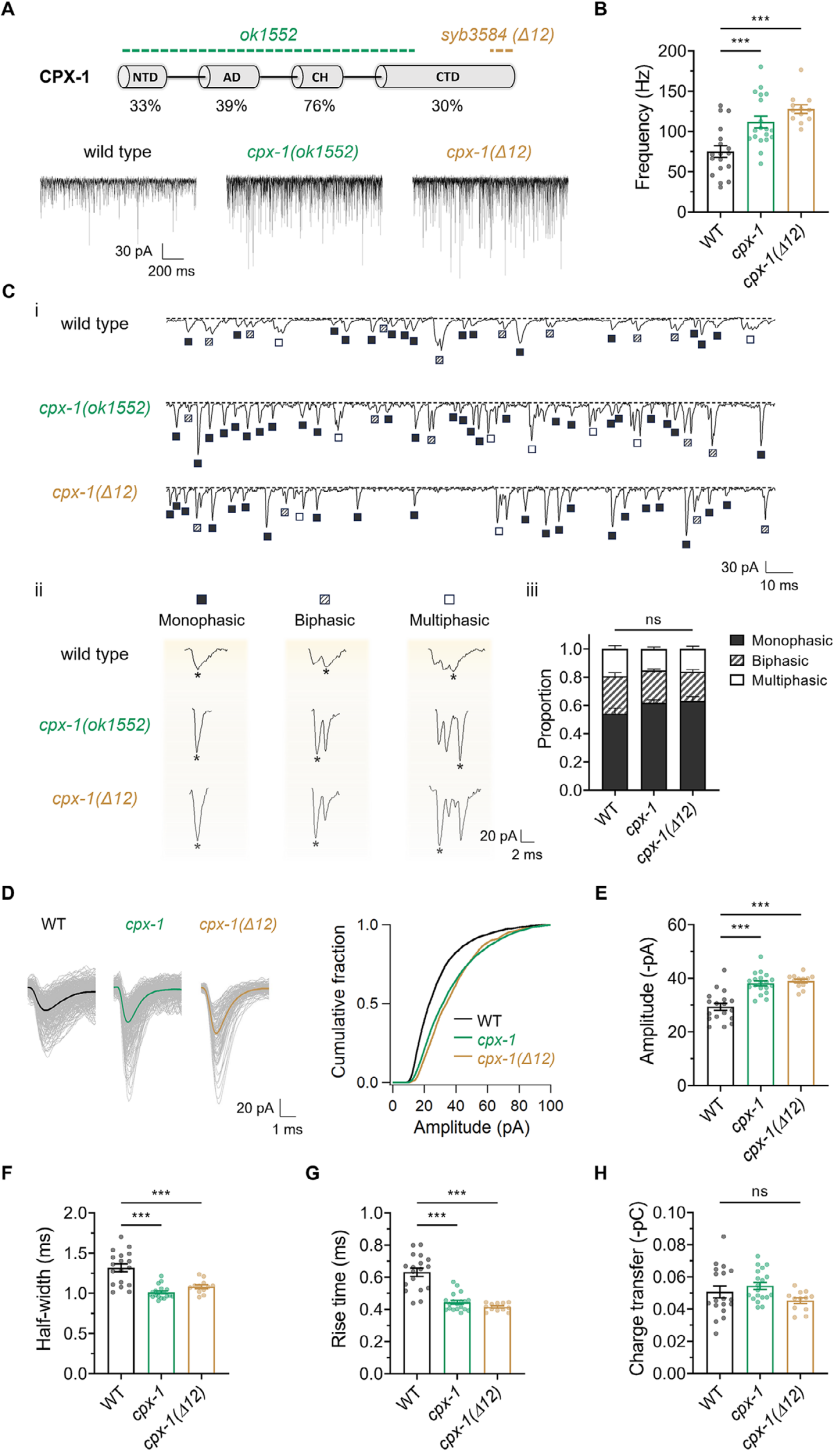
*cpx‐1* mutants display increased amplitude and altered kinetics of spontaneous release. (A) Top panel: Cartoon representation of the *C. elegans* CPX‐1 structure, indicating the domains NTD (N‐terminal domain, 33% identity to mouse complexin 1), AD (accessory domain, 39% identity to mouse complexin 1), CH (central α‐helix, 76% identity to mouse complexin 1), and CTD (C‐terminal domain, 30% identity to mouse complexin 1). Mutation sites of *ok1552* and *syb3584(Δ12)* are labeled with green and brown dashed lines, respectively. Bottom panel: Representative traces of mPSCs recorded from wild‐type (N2), *cpx‐1(ok1552)*, and *cpx‐1(Δ12)* worms. Recordings were made from body wall muscle cells at −60 mV. (B) Quantification of frequency of mPSCs for each strain (WT: *n* = 18; *cpx‐1*: *n* = 19; *cpx‐1(Δ12)*: *n* = 12). (C) Individual spontaneous fusion events in mPSCs were classified into three classes based on waveform morphology: monophasic events (black rectangle), biphasic events (shadow rectangle), and multiphasic events (hollow rectangle). Sample traces of mPSCs from the indicated genotypes with a mixture of the three events (i), representative traces of the three events (ii), and the proportion of the three events in the indicated genotypes (iii). (D) Representative traces (left) and cumulative distributions of the amplitude (right) of monophasic events in each genotype. (E–H) Quantification of amplitude (E), half‐width (F), rise time (G), and charge transfer (H) of monophasic events from the indicated genotypes (WT: *n* = 18; *cpx‐1*: *n* = 19; *cpx‐1(Δ12)*: *n* = 12). One‐way ANOVA was used for comparisons of multiple groups, followed by Dunnett's post hoc test: ***p < 0.001; ns, not significant. All data are presented as the mean ± SEM from three independent experiments.

Among the two complexin genes in *C. elegans*, *cpx‐1* shares higher homology with mammalian complexins and contains four conserved domains: the N‐terminal domain (NTD; 33% identity to mouse), accessory domain (AD; 39% identity to mouse), central α‐helix (CH; 76% identity to mouse), and C‐terminal domain (CTD; 30% identity to mouse) (Figure 1A). Loss of *cpx‐1*, animals display impaired locomotion and increased aldicarb sensitivity [23, 25, 38]. In contrast, *cpx‐2* mutants exhibit no locomotion defects, no changes in aldicarb sensitivity, and no enhancement of the *cpx‐1* phenotype, indicating that CPX‐2 does not function redundantly with CPX‐1 [23, 25]. To dissect the functional diversity of complexin, we focused on CPX‐1 and analyzed two alleles: *cpx‐1(ok1552)*, a null allele with a large deletion spanning the NTD, AD, CH, and part of the CTD; and *cpx‐1(Δ12) (syb3584)* (Figure 1A), a CRISPR‐engineered mutant lacking the C‐terminal 12 residues (131–143) that are critical for clamping function [38].

Consistent with previous findings [38], both *cpx‐1* mutants showed significantly elevated frequency of mPSCs, confirming that CPX‐1 suppresses spontaneous vesicle release (Figure 1A,B). However, unlike the typical characteristics of spontaneous events [25, 39], we found a marked increase in the amplitude of mPSCs at NMJ of *cpx‐1(ok1552)* mutants. In contrast, evoked excitatory postsynaptic currents (EPSCs) were reduced (Figure S2) [23, 25, 38]. A similar amplitude increase of mPSCs was also detected in *cpx‐1(Δ12)* mutants, although evoked‐EPSCs are normal [38]. Elevated mPSC amplitude was reported in *cpx‐1(ok1552)* animals expressing a truncated CPX‐1 lacking the entire CTD (residues 94–143), although no increase in mPSC amplitude was observed in *cpx‐1(ok1552)* animals themselves, either in the presence or absence of Ca^2^^+^ [16]. These results demonstrate that *cpx‐1* essentially regulates the amplitude of spontaneous release, which occurs independently of evoked release.

An intuitive hypothesis is that the elevated mPSC amplitude in *cpx‐1* mutants reflects random overlaps or temporally clustered release events, arising from the markedly increased spontaneous release frequency. To test this, we classified individual spontaneous release events according to their waveform morphology (Figure 1Ci) [40]. Three event classes were distinguished: (1) monophasic events (single peak); (2) biphasic events (two partially overlapping peaks); and (3) multiphasic events (clusters of more than two peaks). These categories were quantified in both wild‐type animals and two *cpx‐1* mutants (Figure 1Cii). Notably, the distribution of event types was indistinguishable between wild‐type and *cpx‐1* mutants (Figure 1Ciii). Thus, the enhanced mPSC amplitude in *cpx‐1* mutants cannot be attributed to synchronized multivesicular fusion resulting from the elevated frequency of spontaneous release.

To minimize the confounding influence of biphasic and multiphasic events on the kinetic analysis of spontaneous release, we restricted our analysis to monophasic events and examined their amplitude and kinetics (Figure 1D). Compared with wild‐type animals, *cpx‐1(ok1552)* mutants showed a pronounced rightward shift in amplitude distribution, resulting in a higher average amplitude (Figure 1D,E). Kinetic analysis further revealed shortened rise time and half‐width in these events (Figure 1F,G), indicating substantially altered mPSC kinetics. By contrast, the total charge transfer remained unchanged (Figure 1H), suggesting that the quantal size—the neurotransmitter content of individual vesicles—was unaffected. Similar changes in amplitude and kinetics were also detected in *cpx‐1(Δ12)* mutants (Figure 1D–H), indicating that CPX‐1 regulates spontaneous release dynamics independently of its role in evoked release. Together, these findings uncover a previously unrecognized in vivo function of CPX‐1 in shaping mPSC amplitude and kinetics, extending beyond its established roles in controlling spontaneous release frequency and synchronized evoked release [23, 24, 25, 26].

*C. elegans* neuromuscular junctions receive both cholinergic and GABAergic inputs, producing mixed excitatory and inhibitory mPSCs under our recording conditions. To determine whether the increased mPSC amplitude observed in *cpx‐1* mutants also extends to inhibitory transmission, we applied 0.5 mM D‐tubocurarine (d‐TBC) to block ionotropic AChRs and isolate miniature inhibitory postsynaptic currents (mIPSCs) [41, 42]. In contrast to excitatory mPSCs, monophasic mIPSCs in *cpx‐1* mutants displayed no differences in amplitude or other kinetics (Figure S1). These findings suggest that CPX‐1 selectively regulates spontaneous mini amplitude at excitatory synapses.

### 2.2 CPX‐1 Regulation of Spontaneous Release Amplitude Is Independent of Ca^2^^+^

Presynaptic vesicle release is tightly regulated by Ca^2^^+^, and complexin is known to act in concert with presynaptic Ca^2^^+^ sensors such as synaptotagmin‐1 to control evoked vesicle release [22, 27]. To test whether the elevated mPSC amplitude in *cpx‐1* mutants reflects Ca^2^^+^‐dependent increases in release frequency—potentially leading to synchronous minis of larger amplitude—we examined spontaneous release across different extracellular Ca^2^^+^ conditions.

**FIGURE 2:**
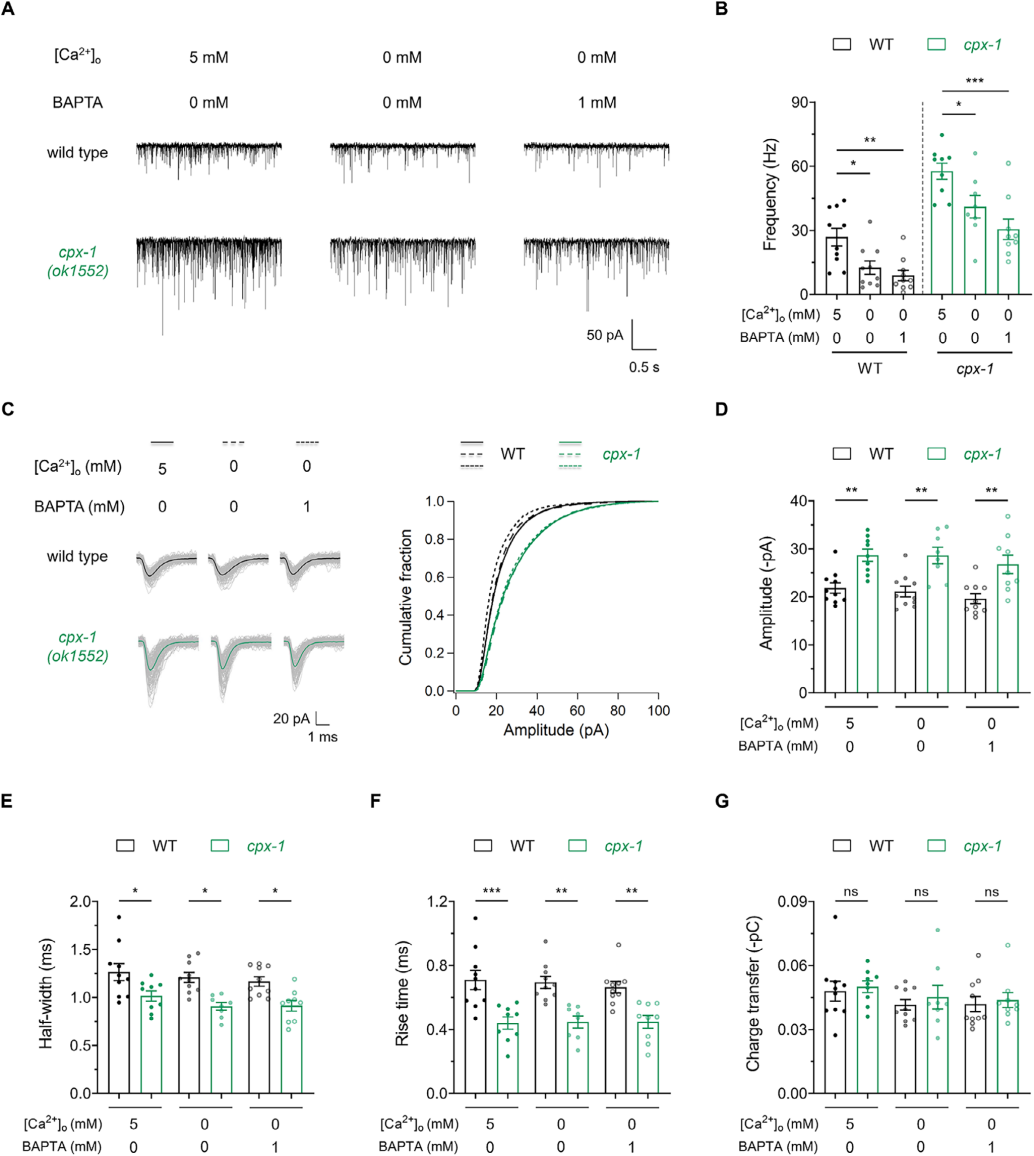
CPX‐1 regulation of spontaneous release amplitude and kinetics is independent of Ca^2^^+^. (A) Representative traces of mPSCs recorded from wild‐type and *cpx‐1 (ok1552)* worms in baths containing 5 mM [Ca^2^^+^]_o_, 0 mM [Ca^2^^+^]_o_, or 0 mM [Ca^2^^+^]_o_ + 1 mM BAPTA‐AM, respectively. (B) Quantification of frequency of monophasic events from the indicated genotypes (WT: *n* = 10; *cpx‐1*: *n* = 9). (C) Representative traces (left) and cumulative distributions of the amplitude (right) of monophasic events. (D–G) Quantification of amplitude (D), half‐width (E), rise time (F), and charge transfer (G) of monophasic events from the indicated genotypes (WT: *n* = 10; *cpx‐1*: *n* = 9). Significant differences were identified by One‐way ANOVA, followed by Tukey's post hoc test: *p < 0.05; **p < 0.01; ***p < 0.001; ns, not significant. Three independent experiments were performed. The error bars represent the SEM.

At high extracellular Ca^2^^+^ (5 mM), *cpx‐1* mutants exhibited a significantly elevated mPSC frequency (Figure 2A,B) [38], along with increased amplitudes and a rightward shift in the amplitude distribution relative to wild type (Figure 2C). Removal of extracellular Ca^2^^+^ (0 mM) reduced mPSC frequency in both genotypes (Figure 2A,B) [38], yet the amplitude in *cpx‐1* mutants remained significantly higher (Figure 2C,D). Pre‐incubation with the Ca^2^^+^ chelator BAPTA‐AM in Ca^2^^+^‐free solution did not diminish the elevated mPSC amplitude observed in *cpx‐1* animals (Figure 2C,D). Analysis of event kinetics revealed that mPSC rise time and half‐width were consistently reduced in *cpx‐1* mutants compared to wild type, regardless of extracellular Ca^2^^+^ concentration or chelation conditions (Figure 2E,F). Total charge transfer remained unchanged across all conditions (Figure 2G).

Together, these findings demonstrate that CPX‐1 regulates mPSC amplitude and kinetics through a mechanism independent of extracellular Ca^2^^+^, potentially via a process distinct from its established role in controlling spontaneous release frequency.

### 2.3 Postsynaptic Restoration of CPX‐1 Rescues Spontaneous Release Amplitude

Increased spontaneous release amplitude and altered kinetics can, in principle, arise from changes in presynaptic vesicle release mechanics–for instance, faster vesicle release dynamics in the absence of complexin [43, 44], or from modifications in postsynaptic receptor abundance or distribution [3, 45], or from a combination of both [46]. We found that loss of CPX‐1 increased mPSC amplitude and altered kinetics without affecting quantal size (Figure 1H) and without dependence on Ca^2^^+^‐regulated high‐frequency release (Figure 2), suggesting that CPX‐1 regulates mPSC amplitude through a mechanism distinct from its presynaptic clamping function. To define the synaptic locus of CPX‐1 action, we tested whether presynaptic or postsynaptic expression was required at the NMJ.

**FIGURE 3:**
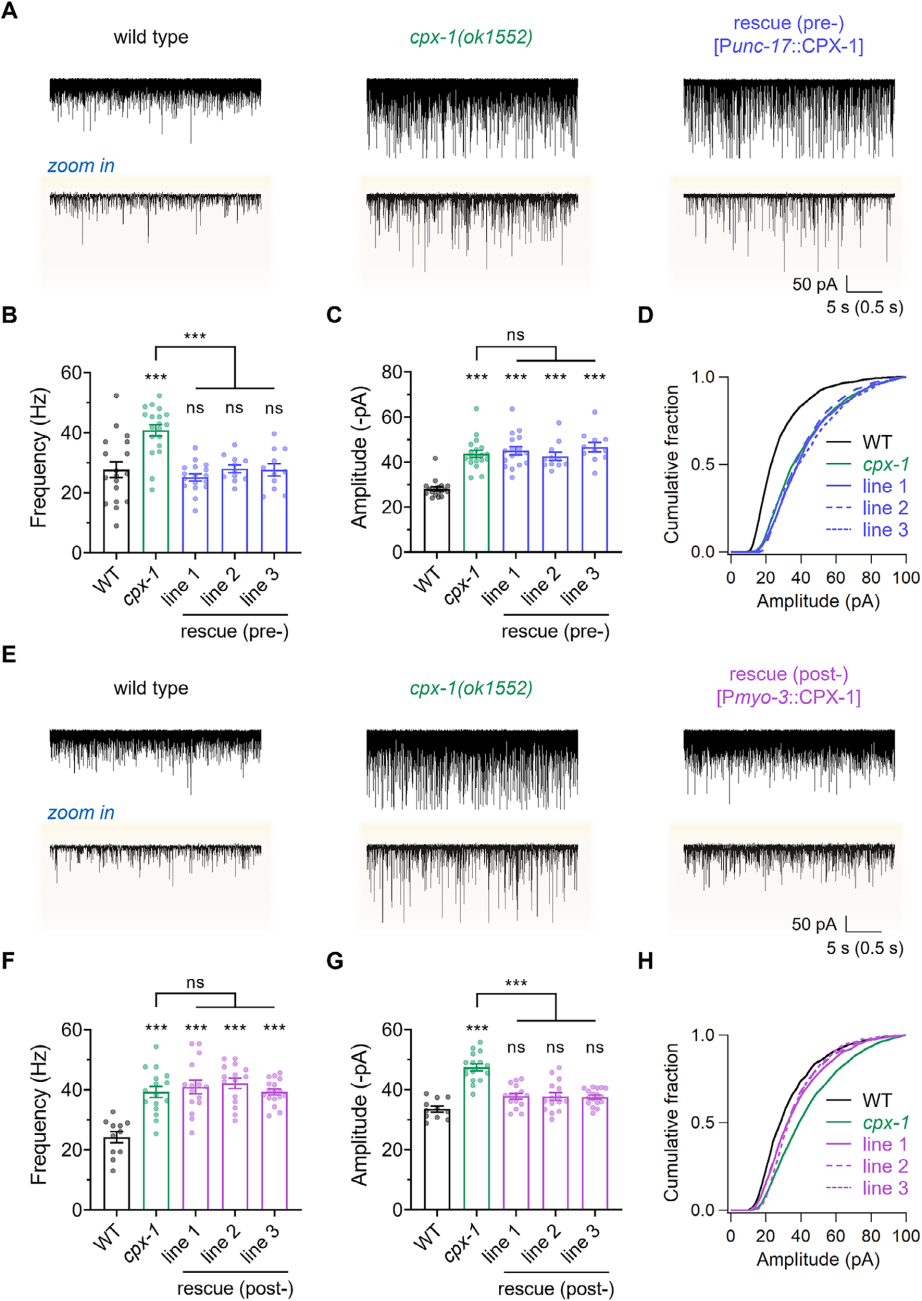
Pre‐ and post‐synaptic CPX‐1 regulate the frequency and amplitude of mPSCs, respectively. (A) Representative traces of mPSCs recorded from wild type, *cpx‐1(ok1552)*, and rescue (presynaptic) strains *cpx‐1(ok1552)*; [P*unc‐17*::CPX‐1] (three independent transgenic lines) at different timescales. (B) Quantification of the monophasic events frequency in mPSCs from the strains mentioned in (A). (C,D) Quantification (C) and cumulative distributions (D) of the amplitude of monophasic events (WT: *n* = 18; *cpx‐1*: *n* = 19; rescue (pre‐) line 1, line 2, line 3: *n* = 17, 11, 11). (E) Representative traces of mPSCs from wild type, *cpx‐1(ok1552)*, and rescue (postsynaptic) strains *cpx‐1(ok1552)*; [P*myo‐3*::CPX‐1] (three independent transgenic lines). (F) Quantification of the monophasic events frequency in mPSCs from the strains mentioned in (E). (G,H) Quantification (G) and cumulative distributions (H) of the amplitude of monophasic events (WT: *n* = 11; *cpx‐1*: *n* = 16; rescue (post‐) line 1, line 2, line 3: *n* = 15, 15, 17). One‐way ANOVA was performed, followed by Tukey's post hoc test: ***p < 0.001; ns, not significant. All data are presented as the mean ± SEM from three independent experiments.

Using the *zxIs6* line, which enables optogenetic stimulation of excitatory synapses [47], we re‐expressed CPX‐1 in cholinergic motor neurons (pre‐rescue, driven by P*unc‐17*). In these transgenic animals, the reduced evoked‐EPSCs observed in *cpx‐1* mutants were fully restored (Figure S2A–C), suggesting that the presynaptic function of CPX‐1 was rescued. The elevated spontaneous release frequency of *cpx‐1* mutants also returned to wild‐type levels (Figure 3A,B), confirming the functional efficacy of the transgene. However, in these animals, mPSC amplitude remained impaired and significantly elevated, indistinguishable from *cpx‐1* null mutants and higher than wild type (Figure 3C,D). Likewise, altered mPSC kinetics (half‐width and rise time) were not rescued (Figure S3A,B). These results demonstrate that presynaptic CPX‐1 regulates evoked release and spontaneous release frequency but does not control mPSC amplitude or kinetics.

This pre‐rescue line functionally mimics a preparation in which postsynaptic muscles lack CPX‐1. The persistence of elevated mPSC amplitude in this context pointed to a postsynaptic role for CPX‐1. Previous work has reported postsynaptic complexin expression across species [31, 32, 48], and in *C. elegans*, *cpx‐1* mRNA is detectable in muscle, albeit at lower levels than in neurons [49, 50]. Additionally, we observed low‐level expression of *cpx‐1* in body wall muscles using the split‐GFP complementary system (Figure S4A–D) [51, 52]. To test its postsynaptic function, we restored CPX‐1 expression in muscle cells (post‐rescue, driven by P*myo‐3*). In this condition, evoked‐EPSCs remained reduced (Figure S2D–F), and spontaneous release frequency was not rescued (Figure 3E,F). Strikingly, however, mPSC amplitude was fully restored to wild‐type levels (Figure 3G,H), and the abnormal kinetics were also rescued (Figure S3C,D).

Together, these results establish that CPX‐1 controls spontaneous release in a compartment‐specific and cell‐autonomous manner: presynaptic CPX‐1 regulates evoked release and spontaneous release frequency, whereas postsynaptic CPX‐1 specifically governs mPSC amplitude and kinetics.

### 2.4 *cpx‐1* Regulates Nicotine‐activated Postsynaptic Currents at Muscle

**FIGURE 4:**
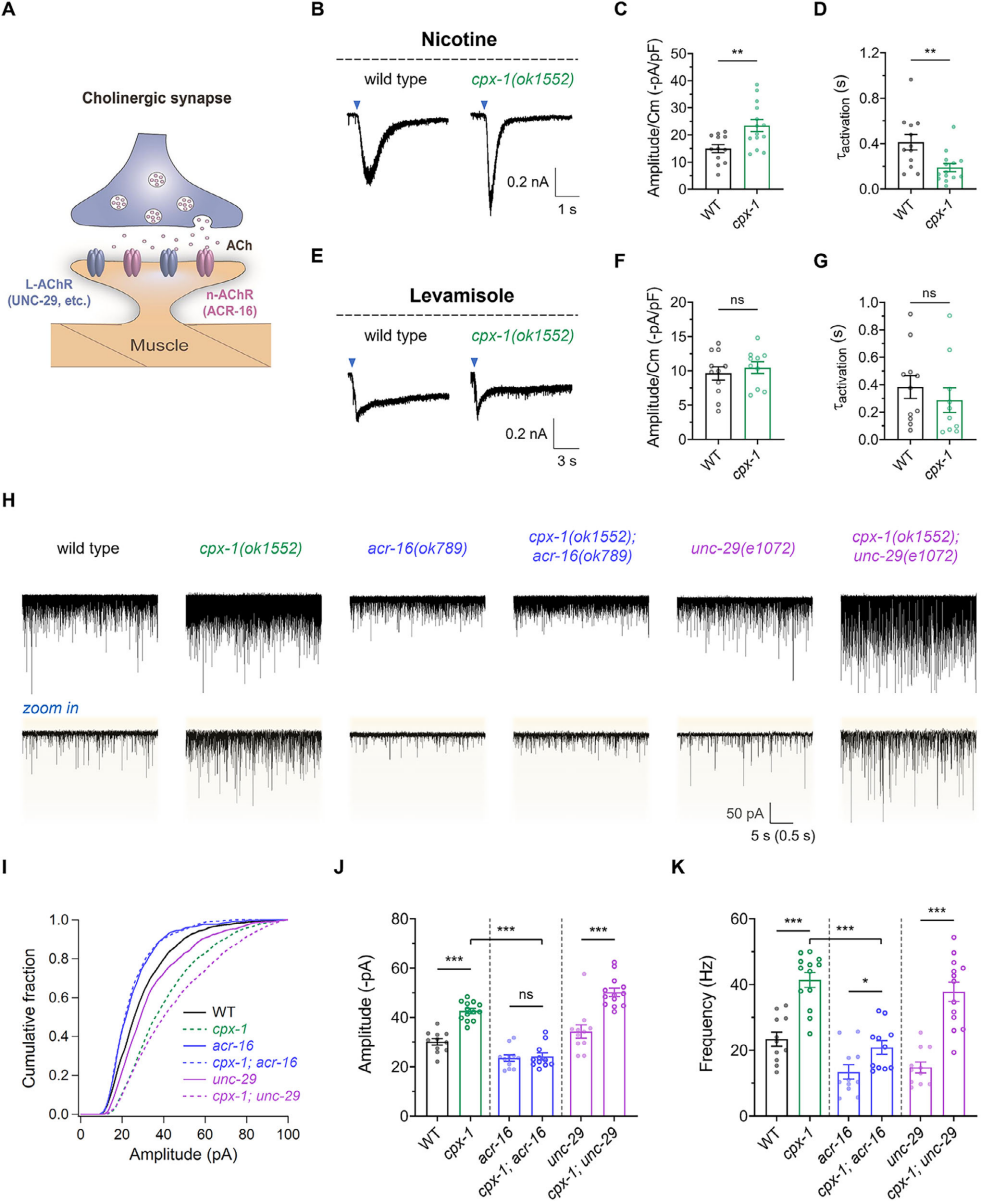
CPX‐1 regulates mPSCs amplitude by modulating the n‐AChR subunit ACR‐16. (A) Cartoon representation of the cholinergic synapse at *C. elegans* NMJ. Cholinergic motor neurons secrete acetylcholine (ACh), which binds and activates ionotropic acetylcholine receptors on muscle cells, the latter of which are divided into two major classes: n‐AChRs (homo‐oligomers composed of ACR‐16 subunits) and L‐AChRs (hetero‐oligomers composed of subunits such as UNC‐29). (B) Representative traces of nicotine (0.1 mM)‐evoked inward currents (I _Nic_) at muscle cells recorded from wild type and *cpx‐1(ok1552)* animals. (C,D) Quantification of current density (C), and activation kinetics (D) of I _Nic_ from the indicated strains (WT: *n* = 12; *cpx‐1*: *n* = 14). (E) Representative traces of levamisole (0.5 mM)‐evoked inward currents (I _Lev_) at muscle cells recorded from wild type and *cpx‐1(ok1552)* animals. (F,G) Quantification of current density (F), and activation kinetics (G) of I _Lev_ (WT: *n* = 11; *cpx‐1*: *n* = 10). (H) Representative traces of mPSCs recorded from the indicated strains at different timescales. (I,J) Cumulative distributions (I) and quantification (J) of the amplitude of monophasic events in mPSCs from the indicated genotypes (WT: *n* = 11; *cpx‐1*: n = 13; *acr‐16*: n = 11; *cpx‐1; acr‐16*: n = 11; *unc‐29*: n = 11; *cpx‐1; unc‐29*: n = 13). (K) Quantification of the monophasic events frequency. Student's *t‐*test was performed for comparisons of two groups, One‐way ANOVA was used for comparisons of multiple groups, followed by Tukey's post hoc test: *p < 0.05; **p < 0.01; ***p < 0.001; ns, not significant. All data are presented as the mean ± SEM from three independent experiments.

The cell‐autonomous regulation of mPSC amplitude by CPX‐1 suggests that *cpx‐1* may directly influence postsynaptic receptor activity or distribution. At the *C. elegans* NMJ, excitatory postsynaptic receptors consist of two pharmacologically and biophysically distinct classes: large‐amplitude, fast‐activating nicotine‐sensitive AChRs (n‐AChRs) and smaller‐amplitude, slowly activating levamisole‐sensitive AChRs (L‐AChRs) (Figure 4A) [42, 53]. To identify which receptor population underlies the *cpx‐1*–dependent changes in spontaneous release amplitude, we recorded pharmacologically evoked whole‐cell currents from body‐wall muscles in vivo. Bath application of nicotine (0.1 mM) in wild‐type animals elicited a robust inward current that activated and inactivated rapidly (I _Nic_) (Figure 4B) [42]. In contrast, perfusion with levamisole (0.1 mM) triggered a smaller, slowly activating inward current (I _Lev_) accompanied by a prominent slowly inactivating steady‐state current (Figure 4E). Strikingly, in *cpx‐1(ok1552)* mutants, the current density of I _Nic_ was significantly enhanced compared to wild type, paralleling the increase in mPSC amplitude (Figure 4B,C). Moreover, I _Nic_ kinetics were altered, exhibiting significantly faster activation (Figure 4B,D), reminiscent of the shortened rise time observed in spontaneous events. By contrast, I _Lev_ current density and kinetics were unchanged in *cpx‐1* mutants (Figure 4E–G).

Together, these findings demonstrate that CPX‐1 specifically regulates nicotine‐activated ACh receptor currents at postsynaptic muscle, thereby identifying n‐AChRs as the postsynaptic receptor population modulated by CPX‐1.

### 2.5 Deletion of *acr‐16* Abolishes the mPSC Amplitude in *cpx‐1* Mutants

Genetically, n‐AChRs are ionotropic receptors assembled from homo‐ or hetero‐oligomeric subunits, including the *acr‐16* gene product, whereas L‐AChRs are built from subunits encoded by genes such as *unc‐29* [42, 54, 55]. To further dissect the receptor subtype underlying CPX‐1–dependent mPSCs amplitude changes, we analyzed spontaneous release properties in *acr‐16* and *unc‐29* mutant backgrounds. Compared to wild‐type animals, *acr‐16(ok789)* single mutants exhibited a pronounced mPSC amplitude reduction (Figure 4H–J), accompanied by a significant decrease in mPSC frequency (Figure 4H,K). In contrast, *unc‐29(e1072)* mutants displayed a reduction in mPSC frequency but no change in amplitude (Figure 4H–K). These results indicate that only *acr‐16* plays a dominant role in determining mPSC amplitude, while both *acr‐16* and *unc‐29* contribute to regulating spontaneous release frequency, consistent with ACR‐16 receptors established role in generating large, fast excitatory events [42].

Crucially, in *cpx‐1(ok1552); acr‐16(ok789)* double mutants, the elevated mPSC amplitude characteristic of *cpx‐1* mutants was completely abolished and reduced to the level observed in *acr‐16* single mutants (Figure 4H–J). By contrast, *cpx‐1(ok1552); unc‐29(e1072)* double mutants retained the increased mPSC amplitude phenotype (Figure 4H–J); in fact, the mPSC amplitude in the double mutant even trended slightly higher than in *cpx‐1* single mutants. Regardless, these data clearly demonstrate that *acr‐16* is strictly required for the CPX‐1–dependent enhancement of mPSC amplitude. Furthermore, deletion of *acr‐16*, but not *unc‐29*, also restored mPSC kinetics (shortened half‐width and rise time) in *cpx‐1* mutants (Figure S5). These findings demonstrate that the *cpx‐1*–dependent increase in mPSC amplitude specifically requires the n‐AChR subunit ACR‐16.

We next examined mPSC frequency across different mutant backgrounds. Loss of *cpx‐1* continued to increase mPSC frequency in *unc‐29* mutants, and the frequency was not significantly different from that observed in *cpx‐1* single mutants (Figure 4K). In contrast, loss of *cpx‐1* in *acr‐16* mutants resulted in a significant reduction in mPSC frequency compared to *cpx‐1* single mutants, although it remained slightly higher than in *acr‐16* single mutants (Figure 4K). These results suggest that the CPX‐1–dependent increase in spontaneous release frequency primarily acts through activation of postsynaptic nAChRs.

To further test this idea, we assessed the receptor dependence of CPX‐1–mediated evoked release. Using the *zxIs6* line [47], we recorded evoked EPSCs across different mutants and found that the evoked currents from excitatory motor neurons are largely mediated by nAChRs, as currents were markedly reduced in *acr‐16(ok789)* mutants but showed little change in *unc‐29(e1072)* mutants (Figure S6A,B) as previously reported [55]. Consistent with previous reports [23, 25, 28], *cpx‐1* mutants exhibited a profound reduction in evoked EPSCs. Interestingly, evoked EPSCs were further reduced in *cpx‐1(ok1552); acr‐16(ok789)* double mutants, but not in *cpx‐1(ok1552); unc‐29(e1072)* double mutants (Figure S6A,B). Total charge transfer followed the same pattern (Figure S6C).

Together, these findings indicate that CPX‐1–mediated regulation of spontaneous release frequency and evoked release also selectively requires ACR‐16–containing nAChRs.

### 2.6 CPX‐1 Mediates Constitutive Exocytosis of ACR‐16

**FIGURE 5:**
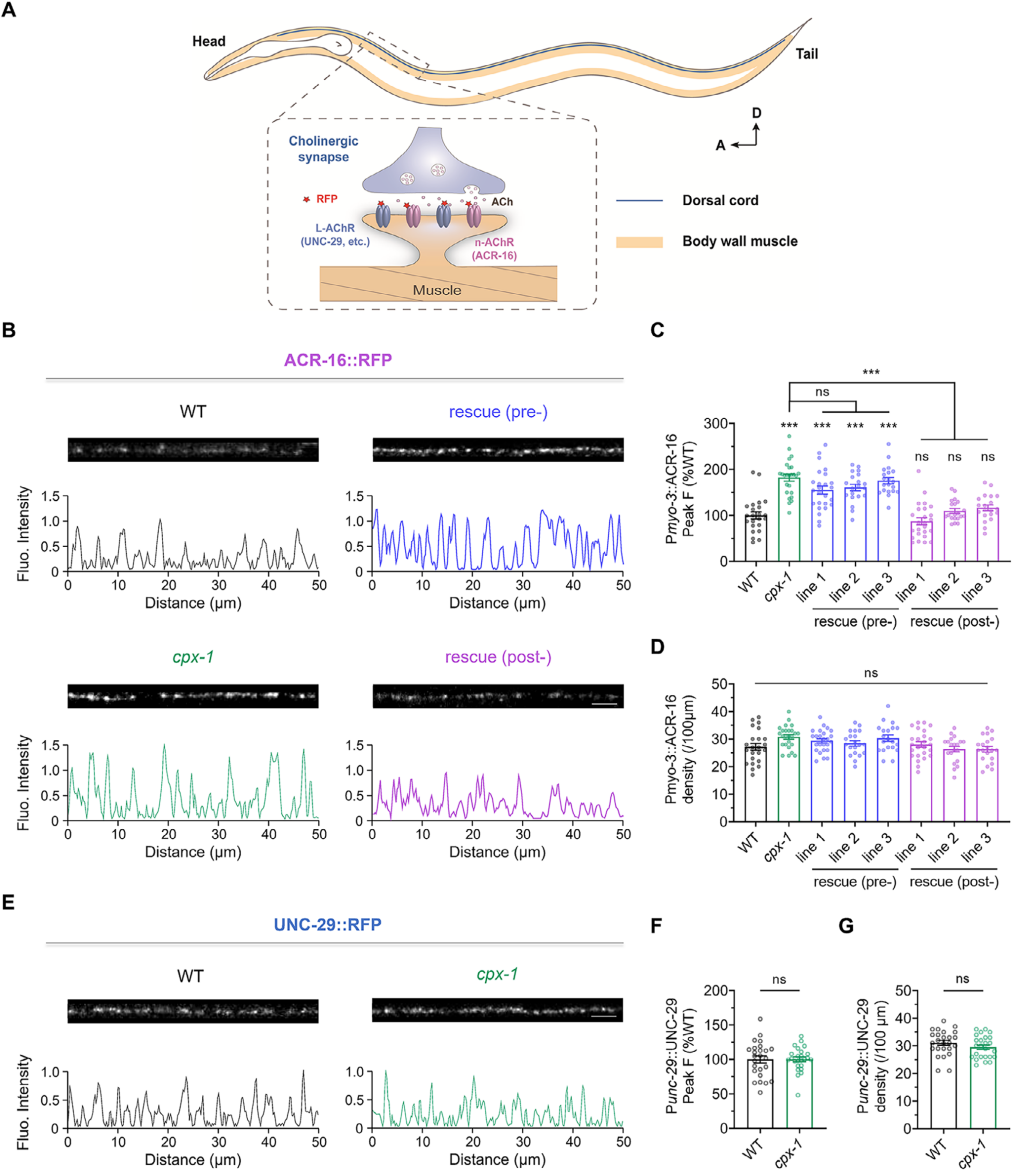
Postsynaptic CPX‐1 mediates constitutive exocytosis of ACR‐16 in body wall muscle. (A) Schematic representation of cholinergic synapse at the NMJ in *C. elegans*. Visualization of endogenous expression of postsynaptic receptors by knocking fluorescent indicator proteins into UNC‐29 or ACR‐16 coding sequences using CRISPR‐Cas9. (B) Confocal micrographs and the fluorescence profile of different genotypes showing ACR‐16::RFP clusters in muscle cells (Scale bar: 5 µm). The fluorescence profiles were all normalized to the WT maximum intensity. Rescue (pre‐) refers to strain *cpx‐1(ok1552)*; [P*acr‐2*::CPX‐1], and rescue (post‐) refers to strain *cpx‐1(ok1552)*; [P*myo‐3*::CPX‐1]. (C,D) Quantification of ACR‐16::RFP puncta maximum fluorescence intensity (C) and synaptic density (D) from the mentioned strains (WT: n = 23; *cpx‐1*: *n* = 25; rescue (pre‐) line 1, line 2, line 3: *n* = 25, 20, 20; rescue (post‐) line 1, line 2, line 3: *n* = 25, 20, 20). (E) Confocal micrographs and the fluorescence profile of WT and *cpx‐1(ok1552)* animals showing UNC‐29::RFP clusters in muscle cells (Scale bar: 5 µm). Both fluorescence profiles were normalized to the WT maximum intensity. (F,G) Quantification of UNC‐29::RFP puncta maximum fluorescence intensity (F) and synaptic density (G) (WT: n = 25; *cpx‐1*: *n* = 25). Student's *t‐*test was performed for comparisons of two groups, One‐way ANOVA was used for comparisons of multiple groups, followed by Tukey's post hoc test: ***p < 0.001; ns, not significant. The error bars represent the SEM. Three independent experiments were performed.

The genetic evidence above demonstrates that the *cpx‐1*–dependent increase in mPSC amplitude and altered kinetics specifically requires the n‐AChR subunit ACR‐16, implicating CPX‐1 in the regulation of postsynaptic receptor exocytosis. To directly assess this, we visualized ACR‐16 trafficking under basal conditions, capturing constitutive receptor exocytosis without exogenous stimulation. We employed a CRISPR‐Cas9–engineered muscle‐specific ACR‐16::RFP knock‐in strain that faithfully reproduces endogenous receptor expression at postsynaptic sites [56]. In this strain, ACR‐16::RFP localized predominantly to postsynaptic regions of motor neurons, forming discrete puncta along neurites (Figure 5A,B). Strikingly, *cpx‐1* null mutants exhibited a robust increase in muscular ACR‐16::RFP fluorescence intensity, with puncta appearing significantly larger and brighter compared to wild‐type animals (Figure 5A–C, Figure S7A), though the puncta density appeared unaffected (Figure 5D).

To pinpoint the site of action, we re‐expressed functional CPX‐1 either in cholinergic motor neurons (presynaptic) or in body‐wall muscles (postsynaptic). Postsynaptic expression fully rescued the aberrant increase in ACR‐16::RFP intensity, whereas presynaptic expression had no effect (Figure 5B,C). This aligns with our electrophysiological data showing that CPX‐1 modulates excitatory mPSC amplitude from the postsynaptic compartment (Figure 3), suggesting that CPX‐1 cell‐autonomously mediates constitutive exocytosis of postsynaptic ACR‐16 receptors.

It is worth noting that fluorescence intensity and density of the L‐AChR subunit UNC‐29::RFP were unchanged in *cpx‐1(ok1552)* mutants relative to wild type (Figure 5E–G, Figure S7B). Loss of *cpx‐1* did not alter the density of ACR‐16::RFP puncta, indicating that synapse number and basal postsynaptic architecture remained intact. Consistently, presynaptic morphology, assessed using the synaptic vesicle marker synaptobrevin (SNB‐1) [57, 58], was normal at both excitatory and inhibitory synapses in *cpx‐1* mutants (Figure S8A–G) in *cpx‐1* mutants, consistent with prior reports [23, 25].

Together, these results reveal that CPX‐1 selectively regulates postsynaptic ACR‐16 exocytosis, without affecting overall synaptic architecture or postsynaptic L‐AChR localization. This receptor‐specific regulation parallels the selective modulation of AMPA receptor exocytosis observed in mammalian hippocampal neurons [31, 59].

### 2.7 CPX‐1 Requires UNC‐31/CAPS for ACR‐16 Exocytosis

In presynaptic vesicle release, complexin interacts with synaptotagmin‐1 to stabilize the SNARE complex and promote synchronous Ca^2^^+^‐triggered neurotransmitter release [22, 27]. Intriguingly, complexin‐dependent postsynaptic receptor exocytosis requires co‐regulators but engages distinct SNARE subunits and Ca^2^^+^ sensors. For example, in cultured hippocampal neurons, the Q‐SNAREs syntaxin‐3 and SNAP‐47 selectively regulate activity‐dependent AMPA receptor exocytosis during LTP, whereas the R‐SNARE synaptobrevin‐2/VAMP2 contributes to both basal and activity‐dependent receptor exocytosis [32]. These findings indicate that complexin‐dependent postsynaptic exocytosis employs a machinery that is molecularly distinct from presynaptic neurotransmission.

**FIGURE 6:**
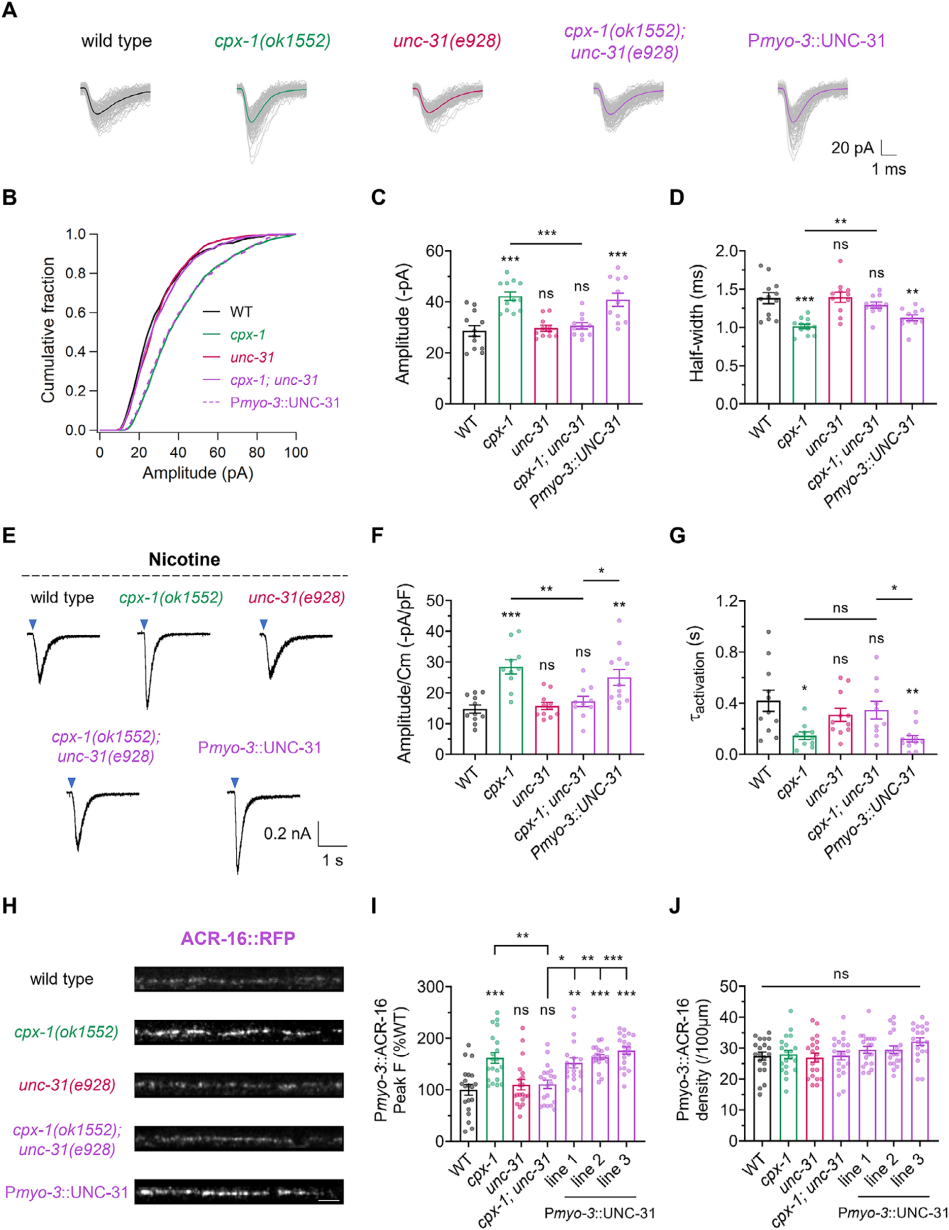
CPX‐1 regulation of ACR‐16 exocytosis is dependent on postsynaptic UNC‐31/CAPS. (A) Representative traces of monophasic events in mPSCs recorded from different genotypes. P*myo‐3*::UNC‐31 refers to the rescue strain *cpx‐1(ok1552)*; *unc‐31(e928)* [P*myo‐3*::UNC‐31]. (B,C) Cumulative distributions (B) and quantification (C) of the amplitude of monophasic events in each strain (WT: *n* = 12; *cpx‐1*: *n* = 12; *unc‐31*: *n* = 11; *cpx‐1; unc‐31*: *n* = 11; P*myo‐3*::UNC‐31: *n* = 11). (D) Quantification of the half‐width of monophasic events from the indicated genotypes. (E–G) Representative traces (E) and quantification of current density (F), and activation kinetics (G) of I _Nic_ recorded from the indicated strains (WT: *n* = 11; *cpx‐1*: *n* = 10; *unc‐31*: *n* = 11; *cpx‐1; unc‐31*: *n* = 10; P*myo‐3*::UNC‐31: *n* = 12). (H–J) Confocal micrographs (H) and quantification of puncta maximum fluorescence intensity (I) and synaptic density (J) of ACR‐16::RFP clusters in muscle cells from different genotypes (WT: *n* = 20; *cpx‐1*: *n* = 20; *unc‐31*: *n* = 20; *cpx‐1; unc‐31*: *n* = 20; P*myo‐3*::UNC‐31 line 1, line 2, line 3: *n* = 20, 20, 20). Scale bar: 5 µm. Significant differences were identified by One‐way ANOVA, followed by Tukey's post hoc test: *p < 0.05; **p < 0.01; ***p < 0.001; ns, not significant. All data are presented as the mean ± SEM from three independent experiments.

We previously reported that complexin‐mediated spontaneous vesicle release at *C. elegans* excitatory synapses requires UNC‐31, a homolog of conserved CAPS (Ca^2^^+^‐dependent activator protein for secretion) primarily implicated in dense‐core vesicle release [38]. To test whether UNC‐31 mediates CPX‐1–dependent postsynaptic regulation, we simultaneously examined mPSC amplitude, nicotine‐evoked receptor currents, and constitutive exocytosis of ACR‐16::RFP across different genotypes. In *unc‐31* mutants, mPSC amplitude and kinetics (half‐width) were indistinguishable from wild‐type animals (Figure 6A–D). Strikingly, deleting *unc‐31* in *cpx‐1(ok1552)* mutants abolished the increased mPSC amplitude and fully rescued the kinetic defects as well (Figure 6A–D). These phenotypes were recapitulated in another *cpx‐1* mutant lacking only the C‐terminal 12 amino acids, *cpx‐1(Δ12)* (Figure S9). This demonstrates that the CPX‐1–driven increase in mPSC amplitude is dependent on UNC‐31. Since *cpx‐1(Δ12)* specifically disrupts spontaneous release without affecting evoked neurotransmission, this result suggests that UNC‐31's regulation of CPX‐1‐dependent postsynaptic currents is independent of CPX‐1's presynaptic role in evoked release. Critically, muscle‐specific expression of UNC‐31 in *cpx‐1; unc‐31* double mutants reinstated the enhanced mPSC amplitude and accelerated kinetics (reduced half‐width) (Figure 6A–D), indicating that UNC‐31 can function at post‐synapses. Similarly, using the split‐GFP complementary system, we observed expression of *unc‐31* in body wall muscles (Figure S4D).

To further test whether CPX‐1 requires UNC‐31/CAPS for ACR‐16 exocytosis, we recorded nicotine‐activated currents from postsynaptic muscles in vivo. Perfusion with nicotine (0.1 mM) in *unc‐31* mutant muscle cells evoked a large, fast‐inactivating inward current, comparable in density and activation kinetics to wild type (Figure 6E–G). This current was markedly smaller and slower than the enhanced I _Nic_ observed in *cpx‐1* mutants. Strikingly, deletion of *unc‐31* in *cpx‐1(ok1552)* mutants restored I _Nic_ to wild‐type levels. Conversely, postsynaptic expression of UNC‐31 in *cpx‐1; unc‐31* double mutants reinstated the elevated I _Nic_ characteristic of *cpx‐1* single mutants (Figure 6E–G). These results demonstrate that CPX‐1 indeed requires UNC‐31/CAPS to drive nicotine‐activated receptor currents at postsynaptic muscles.

To directly test whether UNC‐31 mediates CPX‐1–dependent constitutive exocytosis of ACR‐16, we visualized postsynaptic receptor localization using the muscle‐specific ACR‐16::RFP knock‐in strain. We found that deletion of *unc‐31* in *cpx‐1(ok1552)* mutants abolished the increased ACR‐16::RFP fluorescence observed in *cpx‐1* mutants (Figure 6H–J, Figure S10). However, ACR‐16::RFP puncta intensity along body‐wall muscle neurites remained unchanged in *unc‐31* single mutants compared to the wild type (Figure 6H). Moreover, postsynaptic expression of UNC‐31 in *cpx‐1; unc‐31* double mutants restored fluorescence intensity to the elevated levels observed in *cpx‐1* mutants (Figure 6H–J).

Together, these findings demonstrate that CPX‐1 requires UNC‐31/CAPS to mediate constitutive exocytosis of the postsynaptic n‐AChR subunit ACR‐16, thereby regulating both receptor surface expression and receptor‐driven currents.

### 2.8 CPX‐1 Regulates Activity‐Dependent Exocytosis of ACR‐16

**FIGURE 7:**
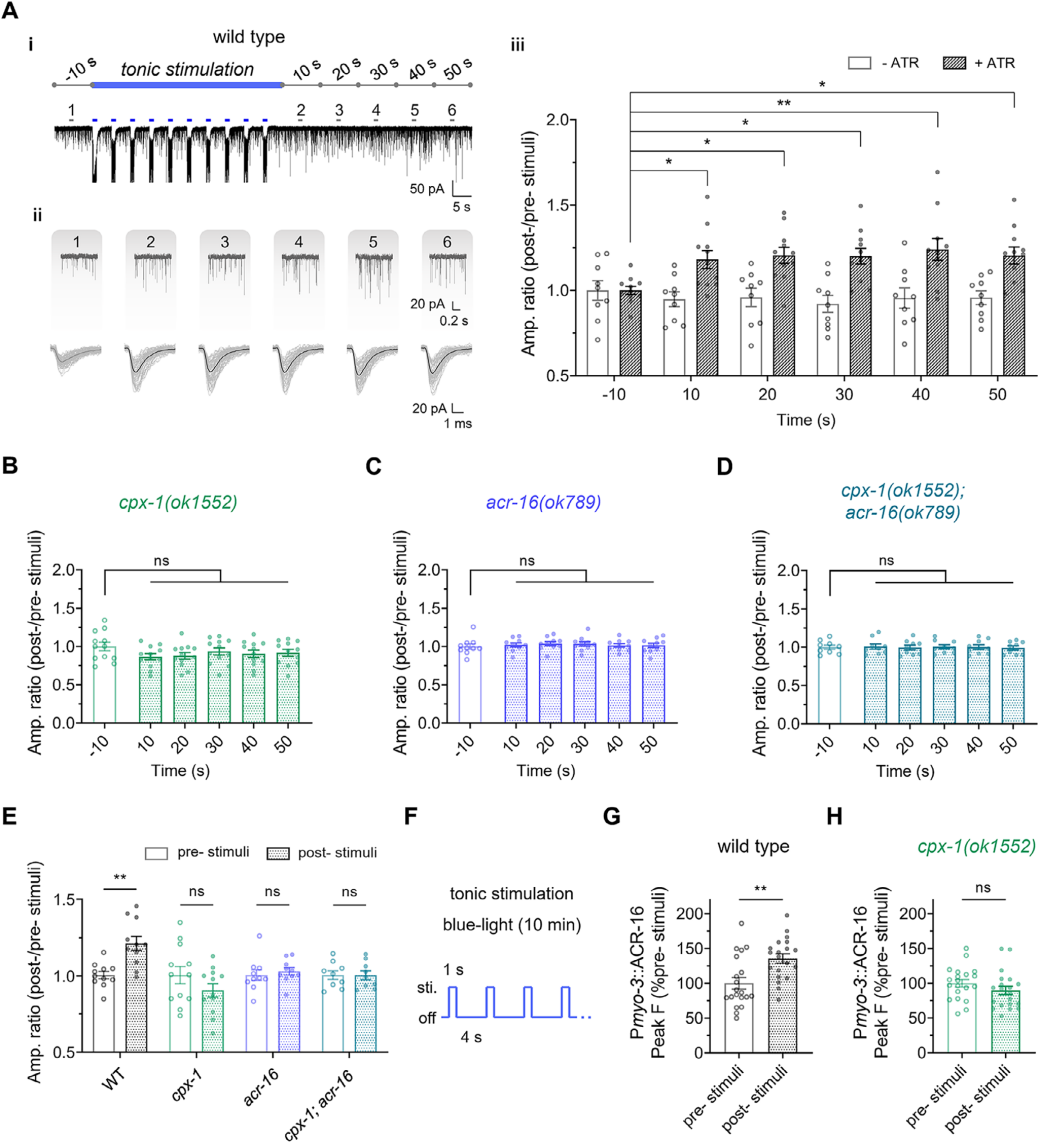
CPX‐1 regulates activity‐dependent ACR‐16 exocytosis induced by optogenetic stimulation. (A) A tonic stimulation program (10 × 1 s pulses at 0.2 Hz) was applied using optogenetics (460 ± 5 nm, 13.75 mW/cm^2^) to induce activity‐dependent synaptic plasticity under the *zxIs6* transgenic background expressing ChR2 in cholinergic neurons. Sample trace of persistent mPSCs before and after stimulation with repeated blue light illumination (blue bars) from wild‐type animals (i). Zoomed‐in views of representative individual minis traces and monophasic events are presented in the gray rounded rectangle, extracted from the indicated regions (lines 1–6) of the upper panel (ii). Ratio between mPSC amplitude post‐ and pre‐tonic stimulation over time (every 10 s) with/without ATR (−ATR: *n* = 9; +ATR: *n* = 11) (iii). Two‐way ANOVA was performed, followed by Sidak's post hoc test. (B–D) Ratio between mPSC amplitude post‐ and pre‐tonic stimulation over time (every 10 s) from indicated genotypes (*cpx‐1*: *n* = 12; *acr‐16*: *n* = 10; *cpx‐1; acr‐16*: *n* = 9). One‐way ANOVA was performed, followed by Dunnett's post hoc test. (E) Ratio between mPSC amplitude post‐ and pre‐tonic stimulation from indicated genotypes. Amplitude statistics were taken for mPSC with a pre‐stimulation length of 10 s and a post‐stimulation length of 50 s, respectively (WT: *n* = 11; *cpx‐1*: *n* = 12; *acr‐16*: *n* = 10; *cpx‐1; acr‐16*: *n* = 9). Student's *t‐*test was performed. (F) Schematic illustration of the extended tonic stimulation pattern. (G,H) Quantification of maximum fluorescence intensity of ACR‐16‐RFP puncta on muscle cells of wild‐type (G) and *cpx‐1(ok1552)* (H) worms before and after stimulation (WT: *n* = 20; *cpx‐1*: *n* = 20). Student's *t* test was performed. *p < 0.05; **p < 0.01; ns, not significant. Three independent experiments were performed and all data are presented as the mean ± SEM.

In cultured hippocampal neurons, postsynaptic complexin regulates activity‐dependent AMPA receptor exocytosis during long‐term potentiation, but not constitutive exocytosis [31, 32]. This prompted us to investigate whether CPX‐1 modulates activity‐dependent exocytosis of ACR‐16. To test this, we applied optogenetic stimulation to cholinergic motor neurons expressing channelrhodopsin‐2 (ChR2) [38]. Using tonic stimulation paradigm, we delivered 10 trains of 1‐s light pulses every 5 s (0.2 Hz) to induce activity‐dependent modulation (Figure 7Ai). In wild‐type animals, tonic stimulation (460 ± 5 nm, 13.75 mW/cm^2^) induced a sustained increase in spontaneous release [38]. Notably, this frequency increase was accompanied by a significant rise in mPSC amplitude in spontaneous release (Figure 7Ai–iii). By contrast, in the absence of all‐trans retinal (ATR), the chromophore for ChR2, light stimulation no longer induced an increase in mPSC amplitude, confirming that the observed enhancement in the amplitude stemmed from increased synaptic activity (Figure 7Aiii). These results suggest that stimulation‐induced spontaneous release plasticity involves both mPSC frequency and amplitude modifications.

This phenomenon is reminiscent of the increase in mPSC amplitude observed in *cpx‐1* mutants. We next asked whether stimulation‐induced mPSC amplitude increase was affected by *cpx‐1*. Remarkably, optical stimulation failed to increase mPSC amplitude in *cpx‐1(ok1552)* mutants, with no significant change in the ratio of average mPSC amplitudes before and after stimulation (Figure 7B). These results indicate that CPX‐1 is also required for activity‐dependent mPSC amplitude. The stimulation‐induced increase in mPSC amplitude was dependent on ACR‐16, as this enhancement was completely abolished in the absence of *acr‐16* (Figure 7C). Furthermore, in *cpx‐1; acr‐16* double mutants, tonic stimulation also failed to increase mPSC amplitude, consistent with the levels observed in single mutants of *cpx‐1* and *acr‐16* (Figure 7D,E). This suggests that CPX‐1 and ACR‐16 operate in the same pathway of activity‐dependent mPSC amplitude elevation.

To further determine whether the observed effects reflect ongoing receptor trafficking rather than developmental compensation in constitutive *cpx‐1* mutants, we used endogenous ACR‐16::RFP fluorescence to directly visualize the effect of optical stimulation on postsynaptic ACR‐16 expression in vivo. Under an extended tonic stimulation paradigm, wild‐type animals showed a significant increase in muscular ACR‐16 fluorescence intensity (Figure 7F,G, Figure S11A), confirming that the increase in mPSC amplitude is due to activity‐dependent ACR‐16 exocytosis. In *cpx‐1* mutants, this fluorescence increase was completely absent (Figure 7H, Figure S11B), indicating that optical stimulation‐induced ACR‐16 exocytosis is CPX‐1‐dependent. These findings, together with our observations of constitutive ACR‐16 exocytosis under spontaneous conditions, underscore the pivotal dual role of postsynaptic CPX‐1. In summary, our results denote that CPX‐1 regulates both constitutive and activity‐dependent exocytosis of ACR‐16.

## 3 Discussion

Our study uncovers a previously unrecognized postsynaptic role for complexin, demonstrating that CPX‐1 directly mediates the exocytosis of postsynaptic n‐AChRs in vivo. This finding refines our understanding of complexin function beyond its canonical role in presynaptic vesicle release [14, 18, 31, 32, 60, 61]. Specifically, we provide the evidence that CPX‐1 cell‐autonomously regulates constitutive ACR‐16 receptor delivery at the NMJ, thereby linking complexin to the basal maintenance of postsynaptic receptor homeostasis. The selective increase in mPSC amplitude reflects enhanced receptor abundance or altered subunit stoichiometry, rather than changes in quantal size, underscoring the importance of local postsynaptic mechanisms for maintaining synaptic strength. The key conceptual advance of this study is that postsynaptic CPX‐1 functions independently of its canonical presynaptic role in clamping spontaneous release. A functional dissociation of CPX‐1 at pre‐ and postsynaptic sites demonstrates that postsynaptic receptor availability can be autonomously regulated, independent of presynaptic release probability.

It is generally accepted that complexin inhibits spontaneous release, and its loss of function may accelerate the release of synaptic vesicles, thereby altering mPSC kinetics and amplitude. Our experimental results show that postsynaptic expression of CPX‐1 alone is sufficient to rescue the enhanced spontaneous release amplitude and mPSC kinetics without changing the release frequency. This supports the notion that CPX‐1 independently regulates receptor abundance or subunit stoichiometry at the postsynapse. Our data also reveal that this postsynaptic role of CPX‐1 is receptor‐selective. Loss of CPX‐1 leads to elevated expression of the excitatory receptor ACR‐16, but not L‐AChRs or GABA receptors. This specificity resembles observations in mammalian neurons where complexin facilitates AMPA—but not NMDA—receptor exocytosis during LTP [31, 32]. By analogy to mammalian AMPA receptor trafficking, CPX‐1 may associate indirectly with UNC‐31/CAPS through specific SNARE partners or accessory factors, such as syntaxin‐3 or SNAP‐47 like proteins, that organize receptor‐selective release sites at the postsynapse. Alternatively, CPX‐1 and UNC‐31/CAPS may influence neuropeptide release from muscle, which in turn modulates synaptic ACR‐16 levels. Although the precise molecular counterparts and signaling pathway in *C. elegans* remain to be defined, such a modular SNARE‐based architecture or an indirect neuromodulation could provide mechanistic frameworks for how CPX‐1 and UNC‐31/CAPS converge to regulate ACR‐16 trafficking without requiring a direct biochemical interaction. Future proteomic analysis and high‐resolution imaging of receptor vesicles may clarify whether complexin directly regulates vesicle maturation, cargo selection, or the final fusion step.

Another major finding is that even under nominally Ca^2^^+^‐free conditions, *cpx‐1* mutants exhibited elevated ACR‐16 abundance and increased mPSC amplitude, indicating a role for CPX‐1 in constitutive, Ca^2^^+^‐independent exocytosis. This differs from observations in isolated mouse hippocampal neurons—where complexin only regulates activity‐dependent receptor exocytosis—suggesting that complexin exhibits multiple regulatory modes. In addition, tonic stimuli‐induced enhanced spontaneous release also requires CPX‐1 in the presence of Ca^2^^+^. These results denote that postsynaptic complexin engages both Ca^2^^+^‐independent and Ca^2^^+^‐dependent pathways to regulate receptor abundance or subunit stoichiometry. Notably, both pathways require UNC‐31/CAPS but differ in Ca^2^^+^ dependence: regulated presynaptic release depends on UNC‐31's Ca^2^^+^‐sensitive function, whereas constitutive postsynaptic trafficking does not, highlighting mechanistic parallels between presynaptic vesicle release and postsynaptic receptor delivery. This suggests that the complexin–CAPS module represents a general exocytic framework employed on both sides of the synapse, albeit with compartment‐specific adaptations.

Despite these advances, several limitations remain in this study. Previous studies have shown that *cpx‐1* and *unc‐31* mRNA are expressed in muscles, but their expression levels are much lower than in presynaptic compartments [49, 50]. While this modest expression of *cpx‐1* and *unc‐31* is sufficient to regulate ACR‐16 trafficking, the underlying mechanisms of how they regulate the muscular vesicle release remain unclear. Regarding the specific postsynaptic mechanisms, questions such as whether ACR‐16 receptor delivery involves vesicular intermediates analogous to those in presynaptic vesicle release are of considerable interest. Experiments to directly visualize such vesicles—using techniques like electron microscopy or live imaging—would greatly facilitate understanding of their morphological characteristics and potential secretory properties. It has been found that UNC‐31/CAPS exhibits little or no protein interaction with CPX‐1 [38]; thus, the requirement for UNC‐31/CAPS in the constitutive, Ca^2^^+^‐independent exocytosis of ACR‐16, as well as the underlying signaling pathway, remains another puzzle. One potential mechanism is that UNC‐31/CAPS may act as a structural cofactor or organizer at postsynaptic sites. Alternatively, UNC‐31/CAPS could employ a regulatory mode at post‐synapses that differs from its presynaptic mode, which require further investigation. Third, the molecular basis for ACR‐16 receptor selectivity of CPX‐1 remains unresolved. Similar to the selective regulation of AMPA receptors by complexin observed in hippocampal neurons in vitro, *cpx‐1* specifically regulates n‐AChRs without affecting levamisole‐sensitive receptors like UNC‐29. The mechanisms underlying this receptor selectivity also constitute a critical and intriguing question. Additionally, how increased ACR‐16 expression levels affect spontaneous release kinetics is another intriguing question. A potential mechanism is that *cpx‐1* mutation may alter the expression of heteromeric nAChR subunits or the stoichiometry between ACR‐16 and these subunits. Addressing these questions will be essential for defining the full postsynaptic exocytic machinery regulated by complexin.

In summary, our findings identify postsynaptic complexin as an essential regulator of receptor exocytosis at excitatory neuromuscular synapses. By coordinating both constitutive and activity‐dependent ACR‐16 exocytosis in a CAPS‐dependent manner, CPX‐1 ensures appropriate receptor density and synaptic strength. The shared reliance of presynaptic and postsynaptic compartments on complexin and CAPS points to a modular exocytic machinery that maintains synaptic homeostasis. These findings expand the functional repertoire of complexin and open new directions for investigating how postsynaptic receptor trafficking is organized, regulated, and integrated into broader mechanisms of synaptic plasticity and stability.

## 4 Experimental Section

### 4.1 Caenorhabditis Elegans Strains

Strains used in this study are listed in Table S1 (Supporting Information). Unless specified differently, the Bristol N2 strain represented the wild type worms. Transgenic strains bearing extrachromosomal arrays (*gaaEx*) were generated by co‐injecting plasmid DNA along with a selectable marker. All *C. elegans* strains were cultured on standard nematode growth medium (NGM) plates that had been seeded with *Escherichia coli* strain OP50, and were consistently maintained at 22°C. Experiments were conducted exclusively using hermaphrodite worms.

### 4.2 Molecular Biology

Constructs used in this study are listed in Table S2 (Supporting Information). All expression plasmids were constructed using the three‐fragment Multisite Gateway system (Invitrogen, Thermo Fisher Scientific, Waltham, MA, USA). The generation of expression clones were achieved through a standard attL‐attR (LR) recombination reaction, whereby the three entry clones (A, B, and C) were recombined into the pDEST R4‐R3 Vector II destination vectors. The entry clone A slot1 was generated using the In‐Fusion method, facilitated by the ClonExpress One Step Cloning Kit (Vazyme, Nanjing). And all entry clones B slot2 and C slot3 were produced through BP recombination reactions. The full‐length cDNA (4126 bp) encoding UNC‐31 and the full‐length gDNA (1193 bp) encoding CPX‐1 were separately cloned into the pDONR221 donor vector, serving as slot2. The fluorescent protein sl2d‐GFP and sl2d‐wCherry were each combined with the pDONR‐P2R‐P3 donor vector to generate slot3 separately.

### 4.3 In Situ Electrophysiology

Dissection and recording followed established protocols [42, 62]. Specifically, hermaphrodite adults aged 1 or 2 days were immobilized using Histoacryl Blue adhesive (Braun, Germany) on a Sylgard (Dow Corning, USA)‐coated cover glass, which was covered with bath solution. Using a glass pipette under a DIC microscope, semi‐fixed worms were given a dorsal dissection. Subsequently, the cuticle flap was flipped and gently adhered to the opposite side using WORMGLU (GluStitch Inc.). Following the removal of the viscera, the intact ventral body muscles were exposed. Anterior body wall muscle cells were patched using borosilicate pipettes (4–6 MΩ resistance; World Precision Instruments, USA) formed by pulling on a P‐1000 micropipette puller (Sutter Instrument). The membrane current was recorded in the whole‐cell mode using EPC‐9 patch clamp amplifier (HEKA, Germany) and PULSE software. The signal is digitally sampled at 10 kHz and then filtered at 2.6 kHz.

The pipette solution contained the following components (in mM): K‐gluconate 115; KCl 25; MgCl_2_ 5; CaCl_2_ 0.1; HEPES 10; BAPTA 1; cAMP 0.5; cGMP 0.5; Na_2_ATP 5; Na_2_GTP 0.5, pH 7.2 with KOH, ∼320 mOsm. cAMP and cGMP were added to preserve the functional activity and longevity of the preparation. The bath solution contained the following components (in mM): NaCl 150; KCl 5; MgCl_2_ 1; CaCl_2_ 5; sucrose 5; glucose 10; and HEPES 15, pH 7.3 with NaOH, ∼330 mOsm. Extracellular bath solutions with different Ca^2^^+^ concentrations were prepared according to different CaCl_2_ concentrations (0 or 5 mM). The zero‐Ca^2+^ solution containing BAPTA is prepared by zero‐Ca^2+^ bath solution with an additional 1 mM of BAPTA added to further chelate and reduce the content of extracellular free Ca^2+^ [39]. When recording mPSCs or evoked‐EPSCs, the muscle cells were clamped at −60 mV. To isolate the mIPSCs, the holding potential was held at −10 mV during recording, and 0.5 mM D‐tubocurarine (d‐TBC) was added to the bath solution to block all acetylcholine receptors [41, 42].

To test the function of acetylcholine receptors on body wall muscles, nicotine and levamisole (concentrations of 0.1 mM and 0.5 mM in the bath solution, respectively) were perfused directly onto patched muscle cells, respectively. All chemicals were purchased from Sigma‐Aldrich unless otherwise specified. Experiments were conducted at room temperature (20–22°C).

### 4.4 Optogenetics

NGM plates were seeded with *E. coli* OP50 supplemented with 500 µM all‐trans retinal (ATR, Sigma) and subsequently stored in the dark at 4°C. Worms at stage L4 was transferred from regular NGM plates to the plates containing ATR. After 24 h of complete dark culture, electrophysiological records were performed. A 460 ± 5 nm LED light source (13.75 mW/cm^2^) was used to stimulate *zxIs6* transgenic strains, evoking postsynaptic currents [47, 63]. This light pulse was triggered by the PULSE software for a duration of 10 ms during the recording of evoked‐EPSCs, unless otherwise specified. In Figure 7, to induce a series of evoked‐EPSCs, a tonic photo‐stimulation protocol was used: 10 pulses of 1‐s duration at 0.2 Hz.

### 4.5 Auxin‐Induced Cell Specific Degradation

In Figures 5 and 6, to image ACR‐16 expressed in muscle cells, worms were grown on auxin plates to degrade ACR‐16::AID::scarlet in the nervous system (P*rab‐3*::TIR1). Specifically, adult worms were transferred to auxin plates and their progeny grown to the L4 stage were analyzed. The auxin plates were prepared by adding 400 mM stock solution (dissolved in ethanol) to the NGM plates, and the final concentration of auxin was 1 mM [64].

### 4.6 Confocal Fluorescence Microscopy

Worms were immobilized by 2.5 mM levamisole (Sigma‐Aldrich) on 2% agarose pads. All fluorescence imaging was done in live L4 hermaphrodites. Fluorescence signals from the strain EN7973 *krSi36[Prab‐3::TIR1::bfp]; kr463[acr‐16::aid::scarlet]* were acquired using an Andor Revolution XD confocal microscope system (Andor Technology plc., Springvale Business Park, Belfast, UK). The system, built on an Olympus IX‐71 inverted microscope (Olympus, Tokyo, Japan) and equipped with a CSU‐X1 (Yokogawa Electric Corporation, Musashino‐shi, Tokyo, Japan) spinning‐disk confocal head, was controlled by Andor IQ 1.91 software. ACR‐16 protein expressed in body wall muscle and labeled with scarlet was imaged using an excitation laser with a wavelength of 561 nm. Fluorescence signals from the strain EN208 *unc‐29(kr208)[unc‐29::tagRFP]*, ZM3030 *nuIs152[Punc‐129::SNB‐1::GFP]* and CZ333 *juIs1[Punc‐25::SNB‐1::GFP]* were performed using a Plan‐Apochromatic 60× objective on an Olympus FV3000 confocal microscope. SNB‐1::GFP or UNC‐29::tagRFP were imaged with a 488 nm or 561 nm excitation wavelength laser, respectively.

For the puncta density and intensity analysis, z‐stacks of the L4 worms of similar size in the mid‐body region were taken in the same conditions. Straightened dorsal nerve cords (100 µm in length) were extracted from the raw images using the “straightened to line function” of ImageJ. The fluorescence intensity along the corresponding cord was calculated using the “plot profile” function of Image J, and puncta numbers were calculated using the “analyze particles” function.

### 4.7 Statistical Analysis

Two‐tailed Student's *t*‐tests were used for comparisons between two groups. One‐way or two‐way ANOVA was applied to assess significant differences among three or more groups. *p* values <0.05 were considered statistically significant: *p* values < 0.05, *p* values < 0.01, and *p* values <0.001 were respectively marked as *, ** and ***. Unless otherwise stated, all data are presented as mean ± SEM. Data analysis and subsequent graphing were performed using Excel (Microsoft, USA), Clampfit (Molecular Devices), ImageJ (National Institutes of Health), Igor Pro (WaveMetrics), MATLAB (MathWorks) and GraphPad Prism 8 (GraphPad Software Inc., USA). For fluorescence imaging and electrophysiology experiments, each recording trace was obtained from an independent animal.

## Author Contributions

S.G. conceived the experiments and wrote the manuscript. Y.W., Y.D., and N.X. performed the experiments and analyzed the data. S.S. contributed to the experiments, discussion and manuscript edition.

## Conflicts of Interest

The authors declare no conflicts of interest.

## Supporting information




**Supporting File**: advs74976‐sup‐0001‐SuppMat.docx.

## Data Availability

The data that support the findings of this study are available in the supplementary material of this article.
