# Supplementary material for: Postsynaptic Complexin Mediates Constitutive Exocytosis of Nicotinic Acetylcholine Receptor
Source: Adv Sci (Weinh). 2026 Mar 26;13(32):e20721. doi: 10.1002/advs.202520721 (PMC13252612; doi:10.1002/advs.202520721)
Supplement: Supplementary file 1 — Supporting File: advs74976‐sup‐0001‐SuppMat.docx. [file ADVS-13-e20721-s001.docx]

**Postsynaptic Complexin Mediates Constitutive Exocytosis of Nicotinic Acetylcholine Receptor**

*Ya Wang ^1^ ^#^, Yixiang Deng ^1 #^, Nan Xia ^1 #^, Shuzo Sugita ^2, 3^, Shangbang Gao ^1, 4^ **

1. Key Laboratory of Molecular Biophysics of the Ministry of Education, College of Life Science and Technology, Huazhong University of Science and Technology, Wuhan 430074, China.

2. Division of Experimental & Translational Neuroscience, Krembil Brain Institute, University Health Network, Ontario, M5T 0S8, Canada.

3. Department of Physiology, Temerty Faculty of Medicine, University of Toronto, Toronto, Ontario, M5S 1A8, Canada.

4. Lead contact.

^#^ These authors contributed equally

* Correspondence: Shangbang Gao, sgao@hust.edu.cn

**
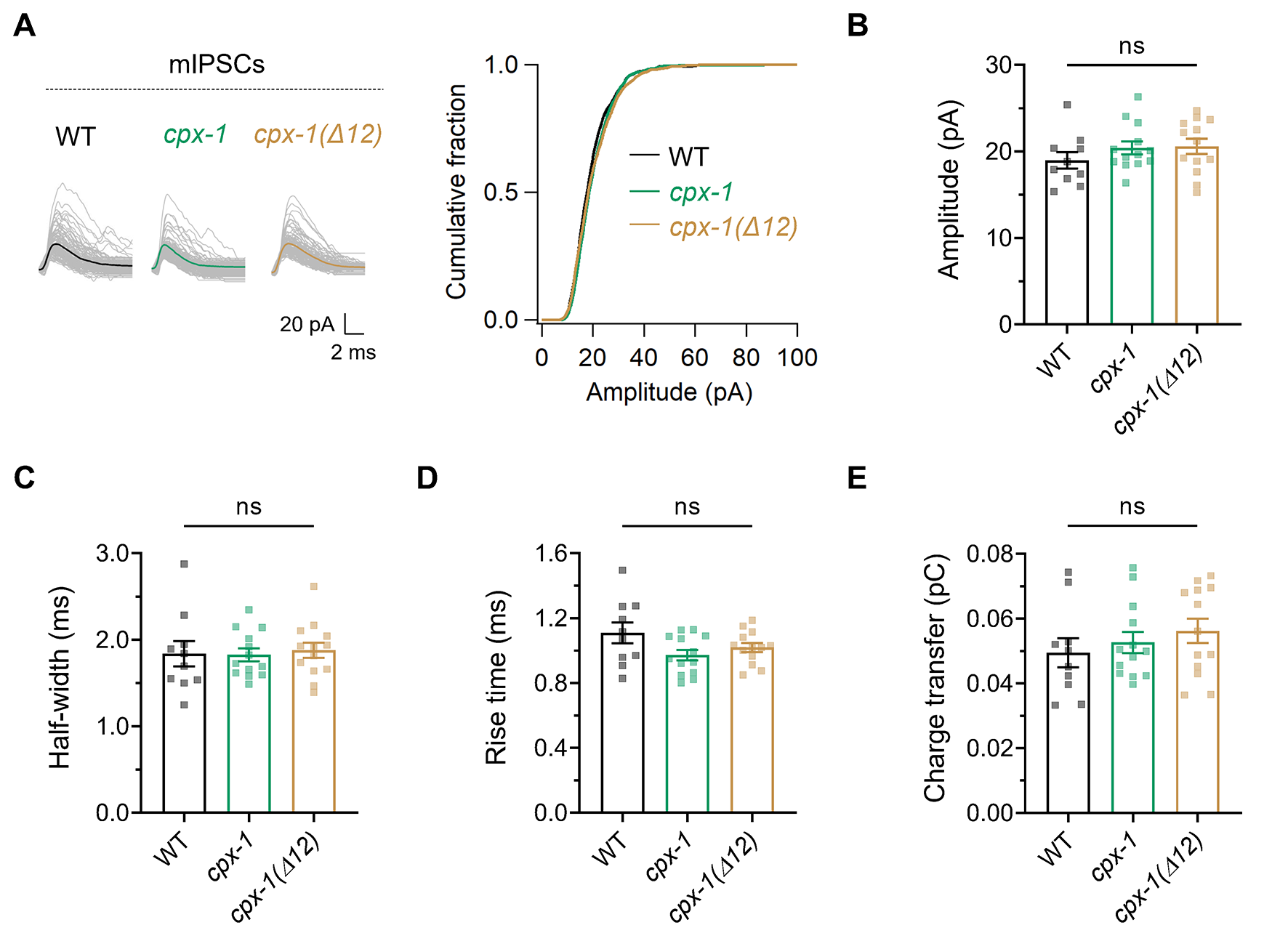
**

**Figure S1.** The *cpx-1* mutants do not display alterations in mIPSCs.

A) Sample traces (left) and cumulative distributions of the amplitude (right) of monophasic events in mIPSCs from WT, *cpx-1(ok1552)*, and *cpx-1(Δ12)* worms. Recordings were made from muscles at −10 mV with the ionotropic acetylcholine receptor blocker D-tubocurarine (0.5 mM).

B**–**E) Quantification of amplitude (B), half-width (C), rise time (D), and charge transfer (E) of monophasic events from the indicated genotypes (WT: *n* = 10; *cpx-1*: *n* = 13; *cpx-1(Δ12)*: *n* = 13).

One-way ANOVA was performed: ns, not significant. All data are presented as the mean ± SEM from three independent experiments.

**
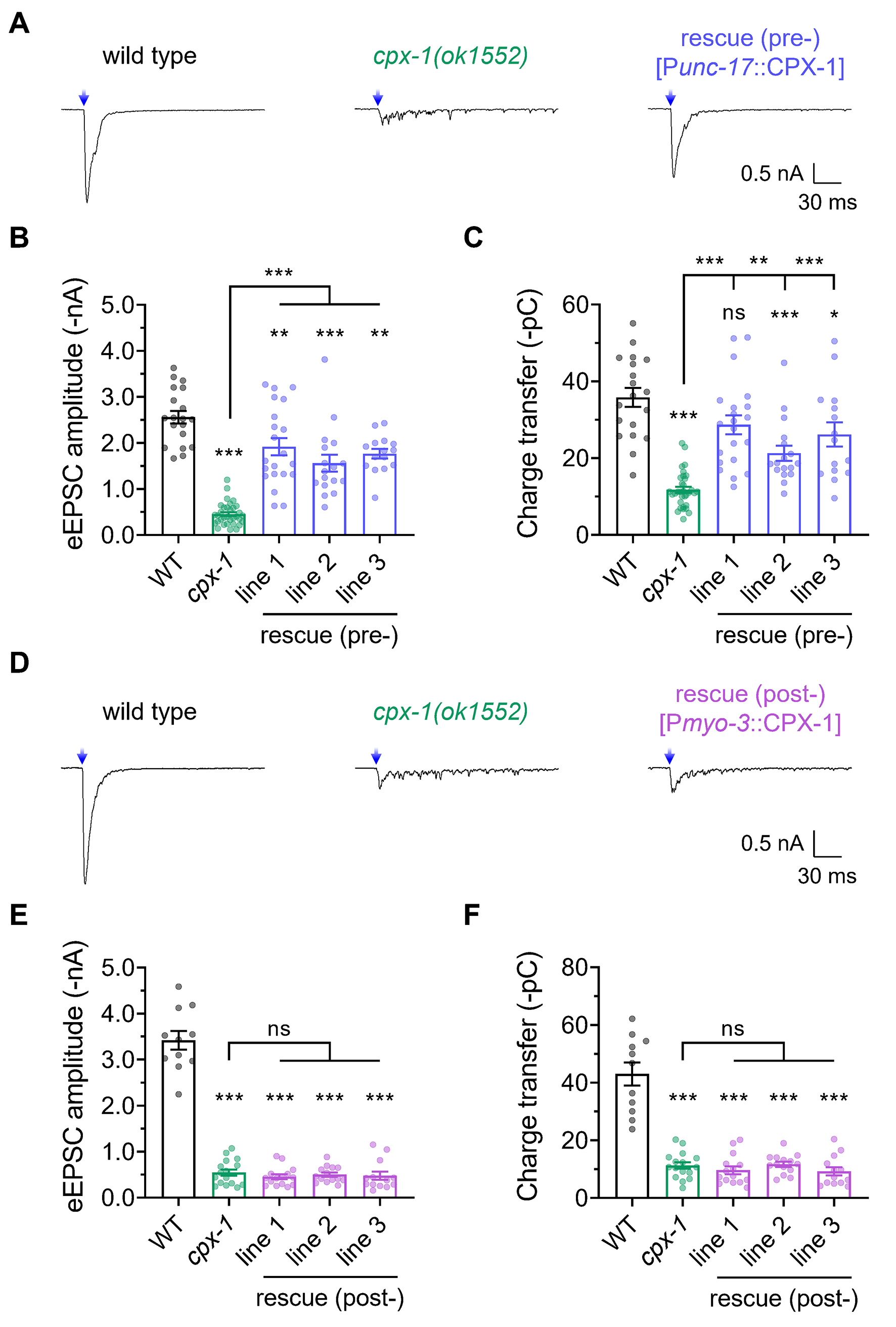
**

**Figure S2.** Presynaptic restoration of CPX-1 rescues evoked-EPSC

A) Representative traces of evoked-EPSC (eEPSC) recorded from the indicated genotypes in the *zxIs6* [P*unc-17*::ChR2::YFP] transgenic background with 10 ms blue light illumination (blue arrow).

B,C) Quantification of eEPSC amplitude (B) and charge transfer (C) in each strain (WT: *n* = 19; *cpx-1*: *n* = 33; rescue (pre-) line 1, line 2, line 3: *n* = 21, 17, 15).

D) Representative traces of eEPSC recorded from the indicated strains in the *zxIs6* transgenic background.

E,F) Quantification of eEPSC amplitude (E) and charge transfer (F) in different genotypes (WT: *n* = 11; *cpx-1*: *n* = 16; rescue (post-) line 1, line 2, line 3: *n* = 14, 14, 13).

Significant differences were identified by One-way ANOVA, followed by Tukey’s post hoc test: **p* < 0.05; ***p* < 0.01; ****p* < 0.001; ns, not significant. The error bars represent the SEM. Three independent experiments were performed.

**
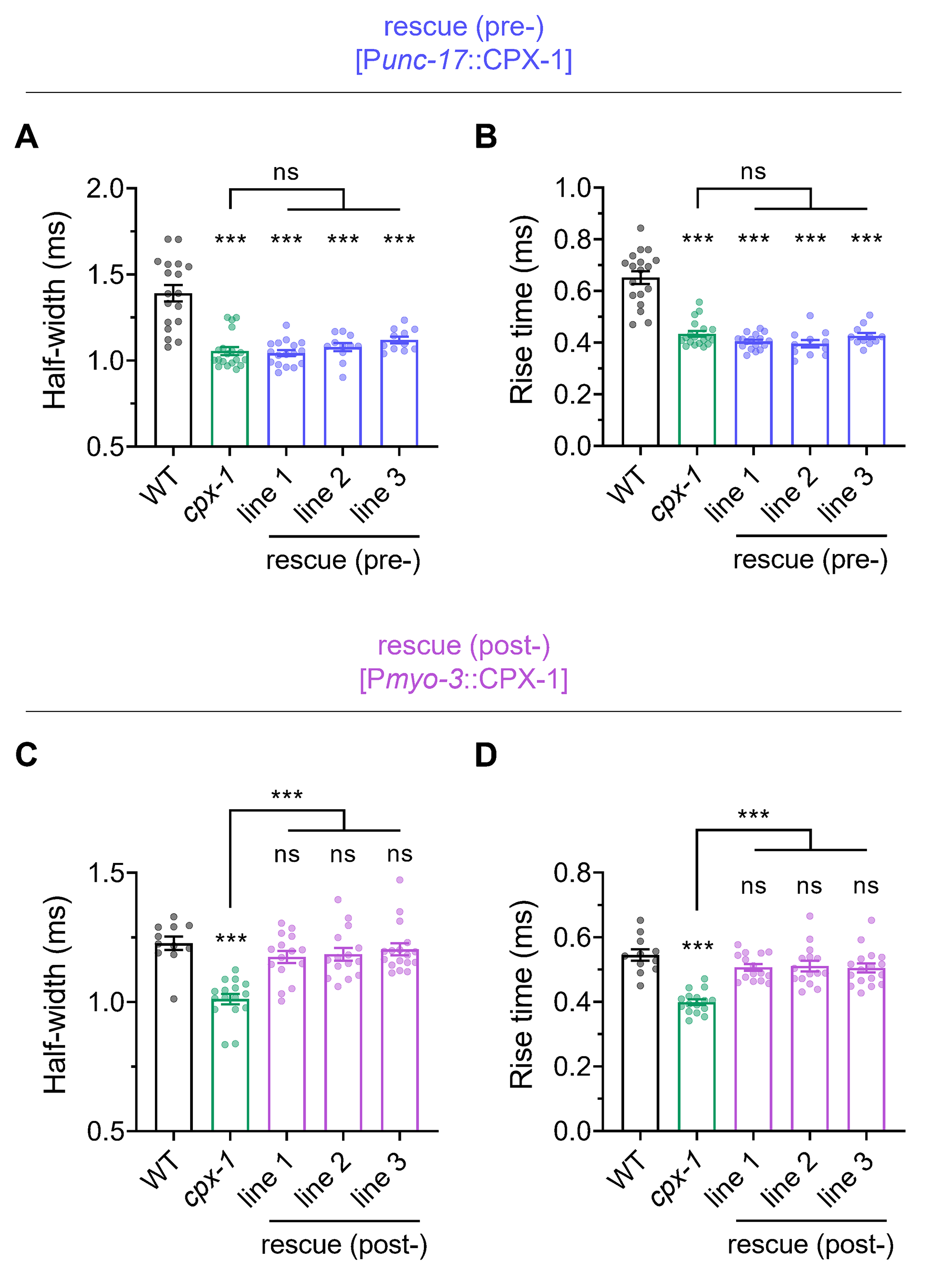
**

**Figure S3.** Postsynaptic restoration of CPX-1 rescues spontaneous release amplitude and kinetics.

A,B) Quantification of half-width (A), and rise time (B) of monophasic events in mPSCs from the mentioned strains (WT: n = 18; cpx-1: n = 19; rescue (pre-) line 1, line 2, line 3: n = 17, 11, 11).

C,D) Quantification of half-width (C), and rise time (D) of monophasic events in mPSCs from the indicated genotypes (WT: n = 11; cpx-1: n = 16; rescue (post-) line 1, line 2, line 3: n = 15, 15, 17).

One-way ANOVA was performed, followed by Tukey’s post hoc test: ****p* < 0.001; ns, not significant. All data are presented as the mean ± SEM from three independent experiments.


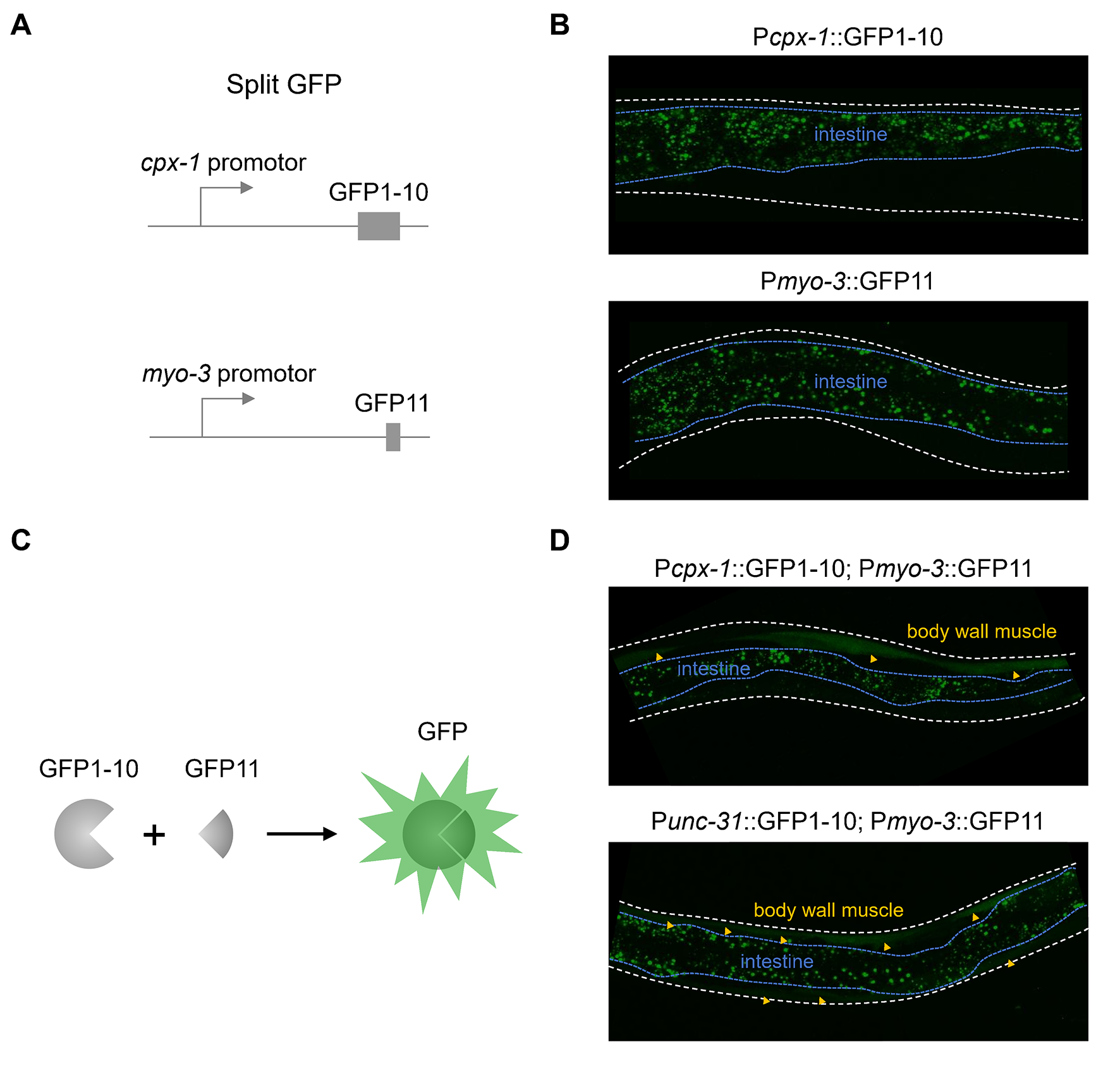


**Figure S4.** Split-GFP system shows the localization of *cpx-1* and *unc-31* in body wall muscles.

A,B) Split GFP1-10 driven by the *cpx-1* promoter or GFP11 driven by the *myo-3* promoter alone did not produce detectable fluorescence in body wall muscles; the blue dashed line outlines intestinal autofluorescence.

C,D) When split GFP1-10 is driven by the *cpx-1* or *unc-31* promoter, it binds GFP11 in body wall muscles to reconstitute GFP and emit green fluorescence, with yellow arrows indicating muscles of typical morphology.

**
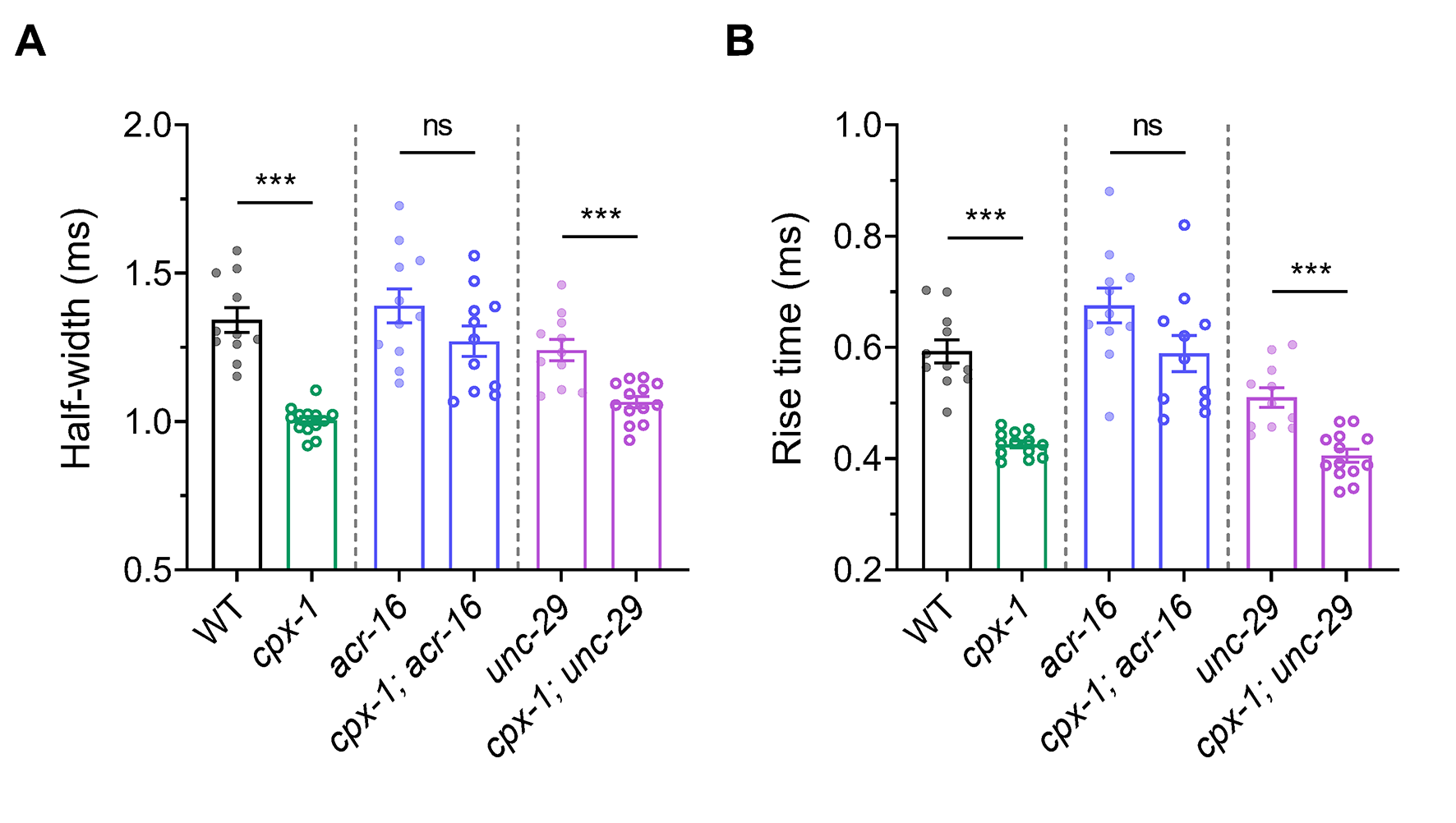
**

**Figure S5.** CPX-1 regulates mPSCs kinetics by modulating the n-AChR subunit ACR-16.

A,B) Quantification of half-width (A), and rise time (B) of monophasic events in mPSCs from the mentioned strains (WT: n = 11; *cpx-1*: n = 13; *acr-16*: n = 11; *cpx-1; acr-16*: n = 11; *unc-29*: n = 11; *cpx-1; unc-29*: n = 13).

Student’s *t* test was performed for comparisons of two groups: ****p* < 0.001; ns, not significant. All data are presented as the mean ± SEM from three independent experiments.

**
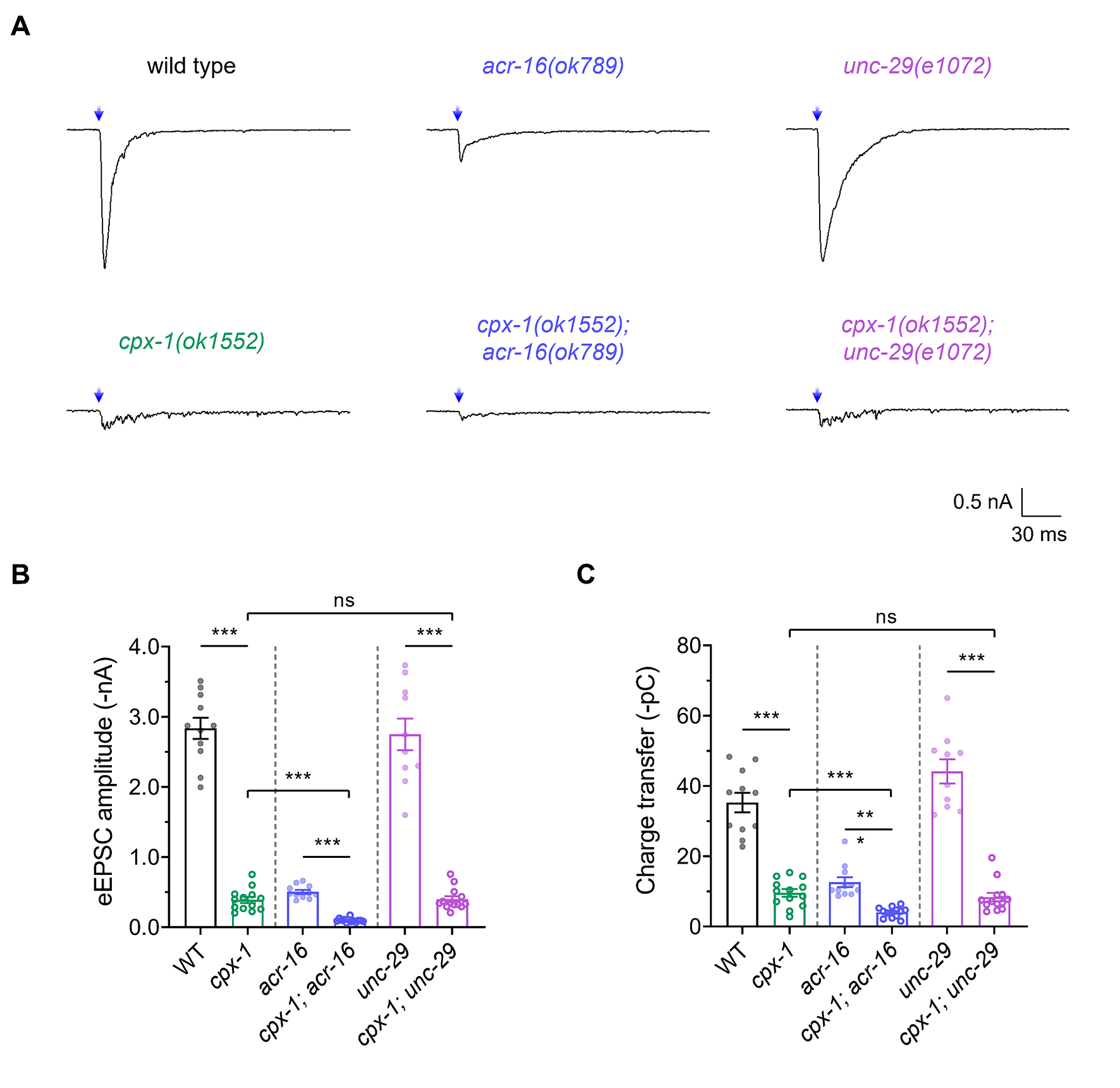
**

**Figure S6.** CPX-1 regulates evoked-EPSC from pre-synapse.

A) Representative traces of eEPSC recorded from the indicated genotypes in the *zxIs6* transgenic background with 10 ms blue light illumination (blue arrow).

B,C) Quantification of eEPSC amplitude (B) and charge transfer (C) in each strains (WT: n = 11; *cpx-1*: n = 13; *acr-16*: n = 11; *cpx-1; acr-16*: n = 13; *unc-29*: n = 10; *cpx-1; unc-29*: n = 13).

Student’s *t* test was performed for comparisons of two groups, One-way ANOVA was used for comparisons of multiple groups, followed by Tukey’s post hoc test: ****p* < 0.001; ns, not significant. Three independent experiments were performed. The error bars represent the SEM.

**
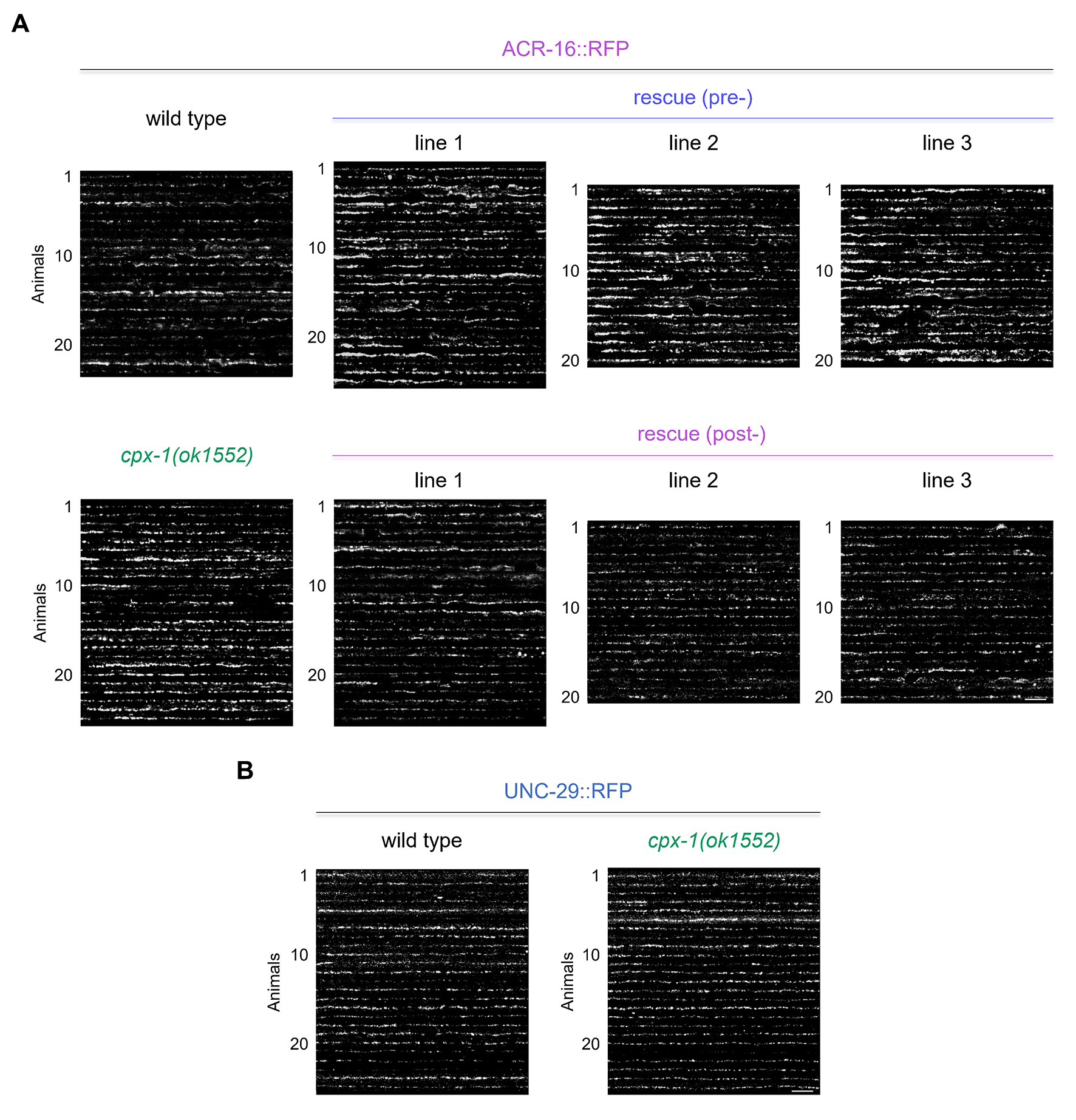
**

**Figure S7.** Postsynaptic CPX-1 regulates ACR-16 abundance in body wall muscle.

A,B) Confocal micrographs showing ACR-16::RFP (A) and UNC-29::RFP (B) clusters in muscle cells from different genotypes. Scale bar: 10 μm.

**
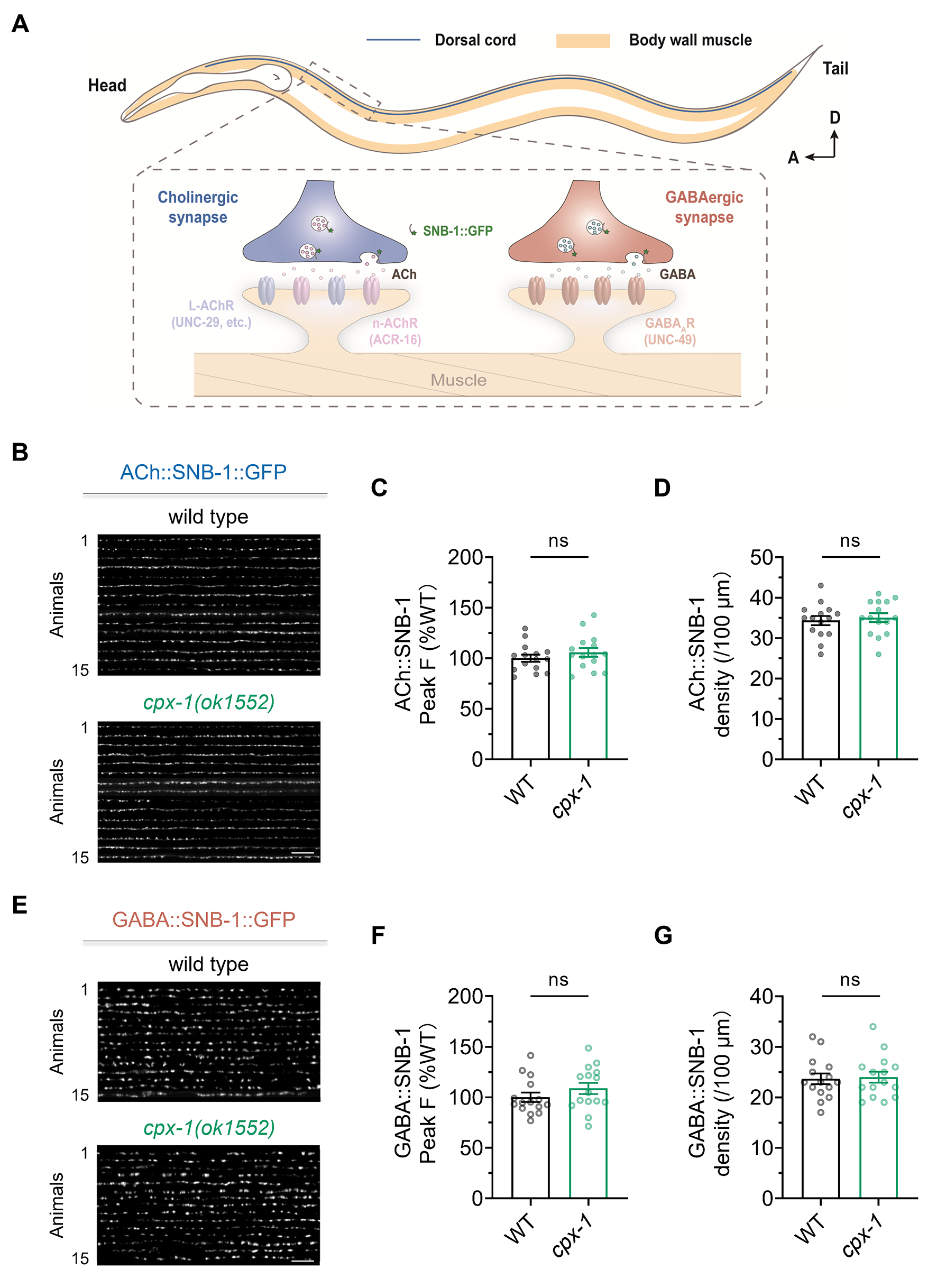
**

**Figure S8.** The *cpx-1* mutation does not affect the overall synaptic architecture of excitatory or inhibitory synapses.

A) Schematic representation of cholinergic and GABAergic synapses at the NMJ in *C. elegans*. Visualization of presynaptic morphology using the synaptic vesicle marker SNB-1::GFP.

B) Confocal micrographs of WT and *cpx-1(ok1552)* animals showing SNB-1::GFP clusters at cholinergic pre-synapses. Scale bar: 10 μm.

C,D) Quantification of ACh::SNB-1::GFP puncta maximum fluorescence intensity (C) and synaptic density (D) from different strains (WT: n = 15; *cpx-1*: n = 15).

E) Confocal micrographs of WT and *cpx-1(ok1552)* animals showing SNB-1::GFP clusters at GABAergic pre-synapses. Scale bar: 10 μm.

F,G) Quantification of GABA::SNB-1::GFP puncta maximum fluorescence intensity (F) and synaptic density (G) (WT: n = 15; *cpx-1*: n = 15).

Significant differences were identified by student’s *t* test: ns, not significant. The error bars represent the SEM. Three independent experiments were performed.


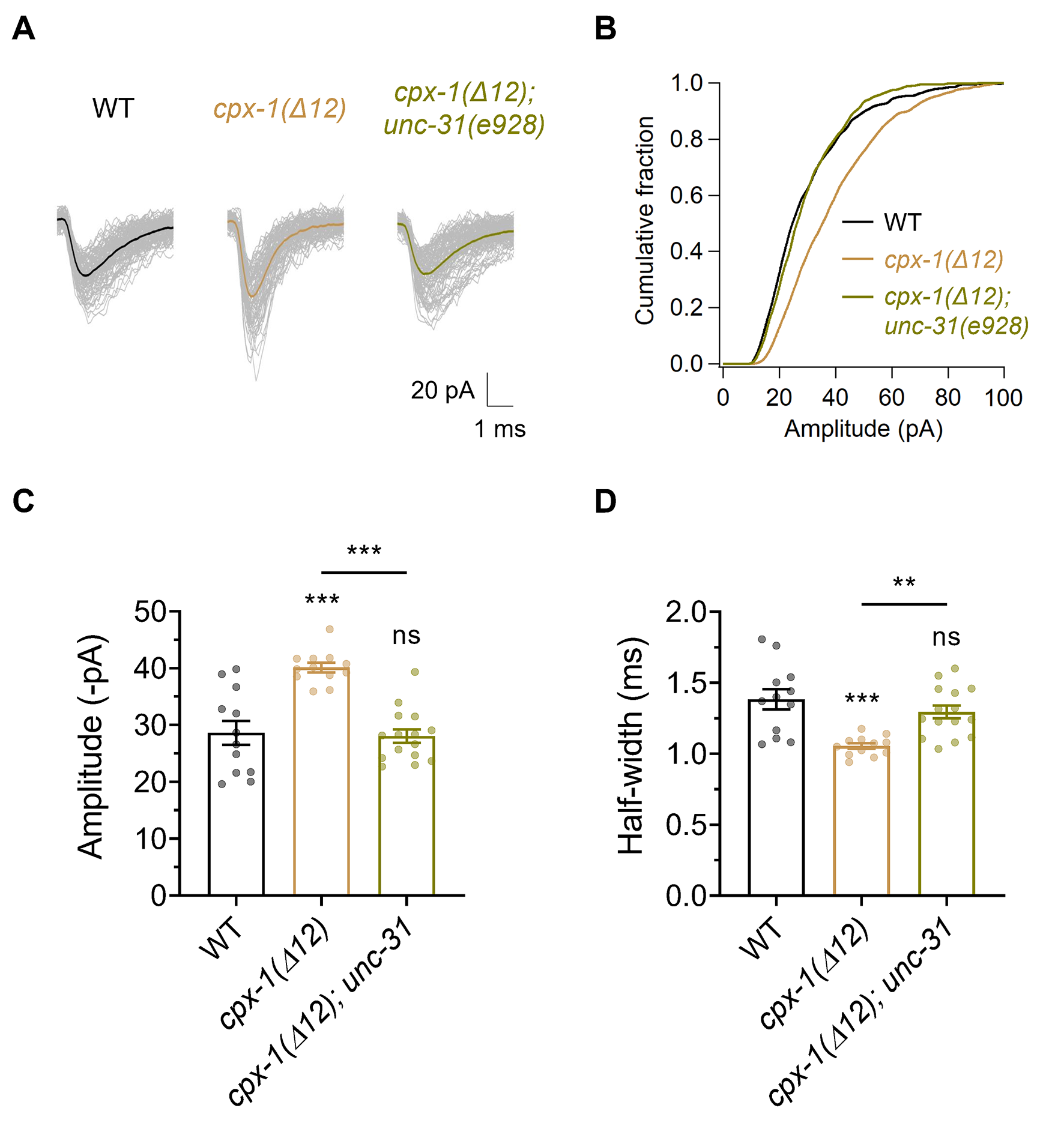


**Figure S9.** The monophasic events amplitude and kinetic alterations caused by the *cpx-1(Δ12)* mutation depend on UNC-31/CAPS

A) Representative traces of monophasic events in mPSCs recorded from wild type, *cpx-1(Δ12)*, and *cpx-1(Δ12); unc-31(e928)* animals.

B,C) Cumulative distributions (B) and quantification (C) of the amplitude of monophasic events in each strain (WT: *n* = 12; *cpx-1(Δ12)*: *n* = 12; *cpx-1(Δ12); unc-31*: *n* = 15).

D) Quantification of the half-width of monophasic events from the indicated strains.

Significant differences were identified by One-way ANOVA, followed by Tukey’s post hoc test: ***p* < 0.01; ****p* < 0.001; ns, not significant. All data are presented as the mean ± SEM from three independent experiments.


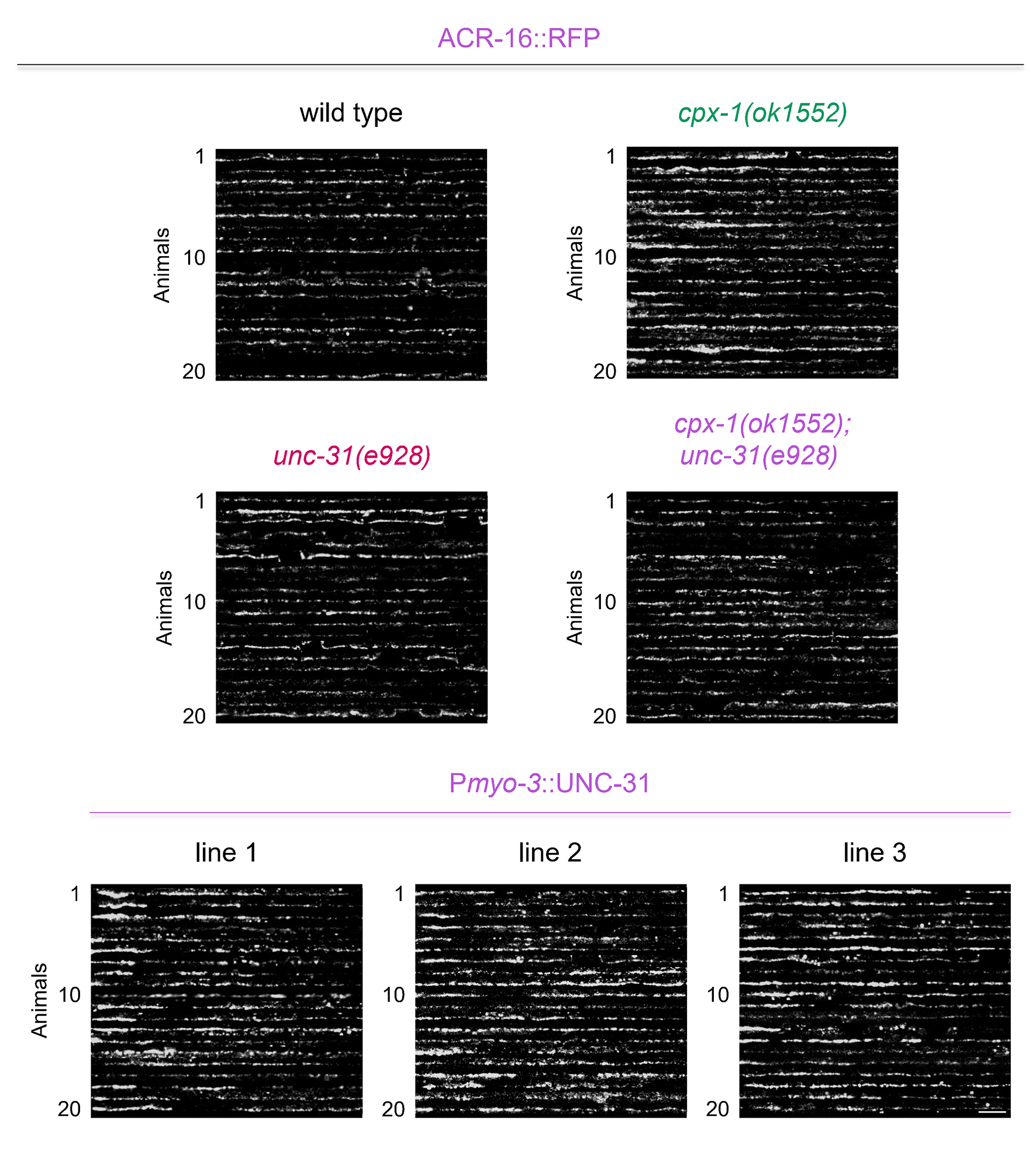


**Figure S10.** CPX-1 regulation of ACR-16 abundance in body wall muscle is dependent on postsynaptic UNC-31/CAPS

Confocal micrographs showing ACR-16::RFP clusters in muscle cells from different genotypes. Scale bar: 10 μm.


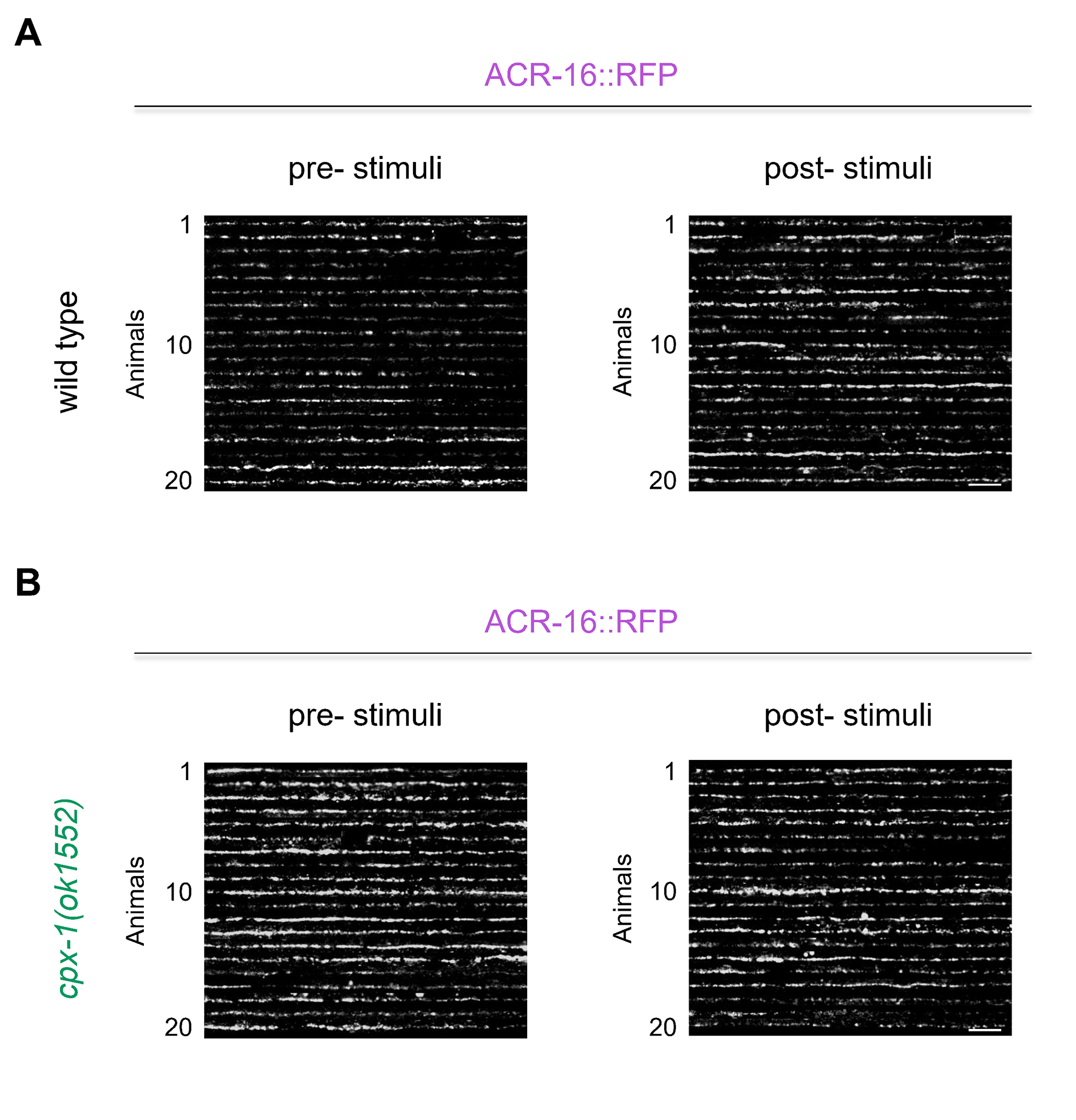


**Figure S11.** CPX-1 regulates activity-dependent ACR-16 exocytosis induced by optogenetic stimulation

A,B) Confocal micrographs showing ACR-16-RFP clusters in wild type (A) and *cpx-1(ok1552)* (B) worm muscle cells before and after tonic stimulation. Scale bar: 10 μm.

**Table S1. *C. elegans* strains used in this study.**

| **Strain name** | **Genotype** |
| --- | --- |
|  | N2 |
| ZX460 | *zxIs6* |
| RB1367 | *cpx-1(ok1552)* |
| UHN70 | *cpx-1(ok1552); zxIs6* |
| PHX3584 | *cpx-1(syb3584)* |
| SGA1043 | *cpx-1(ok1552); zxIs6; gaaEx1295[Punc-17::CPX-1::wCherry; lin-44::GFP] line 1* |
| SGA1494 | *cpx-1(ok1552); zxIs6; gaaEx1295[Punc-17::CPX-1::wCherry; lin-44::GFP] line 2* |
| SGA1502 | *cpx-1(ok1552); zxIs6; gaaEx1295[Punc-17::CPX-1::wCherry; lin-44::GFP] line 3* |
| SGA1157 | *cpx-1(ok1552); zxIs6; gaaEx1428[Pmyo-3::CPX-1::wCherry; lin-44::GFP] line 1* |
| SGA1210 | *cpx-1(ok1552); zxIs6; gaaEx1428[Pmyo-3::CPX-1::wCherry; lin-44::GFP] line 2* |
| SGA1482 | *cpx-1(ok1552); zxIs6; gaaEx1428[Pmyo-3::CPX-1::wCherry; lin-44::GFP] line 3* |
| RB918 | *acr-16(ok789)* |
| SGA979 | *acr-16(ok789); zxIs6* |
| SGA987 | *cpx-1(ok1552); acr-16(ok789); zxIs6* |
| CB1072 | *unc-29(e1072)* |
| SGA983 | *unc-29(e1072); zxIs6* |
| SGA988 | *cpx-1(ok1552); unc-29(e1072); zxIs6* |
| EN7973 | *krSi36[Prab-3::TIR1::bfp]; kr463[acr-16::aid::scarlet]* |
| SGA1246 | *cpx-1(ok1552); krSi36[Prab-3::TIR1::bfp]; kr463[acr-16::aid::scarlet]* |
| SGA1401 | *cpx-1(ok1552); krSi36[Prab-3::TIR1::bfp]; kr463[acr-16::aid::scarlet]; gaaEx1508 [Pacr-2::CPX-1::GFP; lin-44::GFP] line 1* |
| SGA1483 | *cpx-1(ok1552); krSi36[Prab-3::TIR1::bfp]; kr463[acr-16::aid::scarlet]; gaaEx1508 [Pacr-2::CPX-1::GFP; lin-44::GFP] line 2* |
| SGA1486 | *cpx-1(ok1552); krSi36[Prab-3::TIR1::bfp]; kr463[acr-16::aid::scarlet]; gaaEx1508 [Pacr-2::CPX-1::GFP; lin-44::GFP] line 3* |
| SGA1380 | *cpx-1(ok1552); krSi36[Prab-3::TIR1::bfp]; kr463[acr-16::aid::scarlet]; gaaEx1502 [Pmyo-3::CPX-1::GFP; lin-44::GFP] line 1* |
| SGA1484 | *cpx-1(ok1552); krSi36[Prab-3::TIR1::bfp]; kr463[acr-16::aid::scarlet]; gaaEx1502 [Pmyo-3::CPX-1::GFP; lin-44::GFP] line 2* |
| SGA1487 | *cpx-1(ok1552); krSi36[Prab-3::TIR1::bfp]; kr463[acr-16::aid::scarlet]; gaaEx1502 [Pmyo-3::CPX-1::GFP; lin-44::GFP] line 3* |
| EN208 | *unc-29(kr208)[unc-29::tagRFP]* |
| SGA946 | *cpx-1(ok1552); unc-29(kr208)[unc-29::tagRFP]* |
| ZM3030 | *nuIs152[Punc-129::SNB-1::GFP; Pttx-3::mrfp]* |
| SGA1360 | *cpx-1(ok1552); nuIs152[Punc-129::SNB-1::GFP; Pttx-3::mrfp]* |
| CZ333 | *juIs1[Punc-25::SNB-1::GFP]* |
| SGA1409 | *cpx-1(ok1552); juIs1[Punc-25::SNB-1::GFP]* |
| DA509 | *unc-31(e928)* |
| UHN73 | *cpx-1(ok1552); unc-31(e928)* |
| SGA1383 | *cpx-1(ok1552); unc-31(e928); gaaEx1503[Pmyo-3::UNC-31::GFP; lin-44::GFP]* |
| SGA1404 | *unc-31(e928); krSi36[Prab-3::TIR1::bfp]; kr463[acr-16::aid::scarlet]* |
| SGA1387 | *cpx-1(ok1552); unc-31(e928); krSi36[Prab-3::TIR1::bfp]; kr463[acr-16::aid::scarlet]* |
| SGA1396 | *cpx-1(ok1552); unc-31(e928); krSi36[Prab-3::TIR1::bfp]; kr463[acr-16::aid::scarlet]; gaaEx1507[Pmyo-3::UNC-31::GFP; lin-44::GFP] line 1* |
| SGA1485 | *cpx-1(ok1552); unc-31(e928); krSi36[Prab-3::TIR1::bfp]; kr463[acr-16::aid::scarlet]; gaaEx1507[Pmyo-3::UNC-31::GFP; lin-44::GFP] line 2* |
| SGA1488 | *cpx-1(ok1552); unc-31(e928); krSi36[Prab-3::TIR1::bfp]; kr463[acr-16::aid::scarlet]; gaaEx1507[Pmyo-3::UNC-31::GFP; lin-44::GFP] line 3* |
| SGA1402 | *zxIs6; krSi36[Prab-3::TIR1::bfp]; kr463[acr-16::aid::scarlet]* |
| SGA1408 | *cpx-1(ok1552); zxIs6; krSi36[Prab-3::TIR1::bfp]; kr463[acr-16::aid::scarlet]* |
| SGA1533 | *N2; gaaEx1557[Pcpx-1::GFP1-10; Pmyo-3::GFP11; Pmyo-2::RFP]* |
| SGA1534 | *N2; gaaEx1557[Punc-31::GFP1-10; Pmyo-3::GFP11; Pmyo-2::RFP]* |
| SGA1535 | *N2; gaaEx1559[Pcpx-1::GFP1-10; Pmyo-2::RFP]* |
| SGA1536 | *N2; gaaEx1560[Pmyo-3::GFP11; Pmyo-2::RFP]* |
| SGA1548 | *N2; gaaEx1566[Punc-31::GFP1-10; Pmyo-2::RFP]* |

**Table S2. List of the constructs used in this study.**

| **Construct** | **Information** |
| --- | --- |
| SG2 | *pDONR221* |
| SG4 | *PDESTR4-R3II* |
| SG5 | *Plin-44::GFP* |
| SG321 | *Pmyo-2::RFP* |
| SG727 | *Punc-17::CPX-1::wCherry* |
| SG730 | *Pmyo-3::CPX-1::wCherry* |
| SG731 | *Pacr-2::CPX-1::GFP* |
| SG732 | *Pmyo-3::CPX-1::GFP* |
| SG733 | *Pmyo-3::UNC-31::GFP* |
| SG734 | *Pcpx-1::GFP1-10* |
| SG735 | *Punc-31::GFP1-10* |
| SG736 | *Pmyo-3::GFP11* |
